# Low-intensity pulsed ultrasound for peripheral nerve regeneration: mechanobiological mechanisms and translational potential

**DOI:** 10.1186/s12967-025-07638-1

**Published:** 2025-12-30

**Authors:** Nhat Dang Huy Nguyen, Ching-Hsiang Fan, Shun-Ho Huang, Wentai Liu, Peng-Ting Chen, Yuan-Yu Hsueh

**Affiliations:** 1 Biomedical Engineering Department, National Cheng Kung University, No. 1, University Road, Tainan, Taiwan; 2 Center of Orthopaedic and Plastic Surgery, Hue Central Hospital, Hue, 490000 Vietnam; 3 Center for Transformative Bioelectronic Medicine, National Cheng Kung University, No. 1, University road, Tainan, Taiwan; 4 Department of Bioengineering, University of California, Los Angeles, CA 90095 USA; 5 International Institute of Medical Device Innovation, National Cheng Kung University, No. 1, University Road, Tainan, Taiwan; 6 Division of Plastic and Reconstructive Surgery, Department of Surgery, National Cheng Kung University Hospital, College of Medicine, National Chenkg Kung University, No. 138, Sheng-Li road, Tainan, Taiwan; 7 Department of Physiology, College of Medicine, National Cheng Kung University, No. 1, University Road, Tainan, Taiwan

**Keywords:** Peripheral nerve injuries, Low-intensity pulsed ultrasound, Schwann cells, Nerve regeneration, Signal transduction, Neural stem cells, Ultrasonic therapy

## Abstract

**Background:**

Peripheral nerve injury (PNI) remains a major clinical challenge, often resulting in incomplete functional recovery despite advances in microsurgical repair. Low-intensity pulsed ultrasound (LIPUS) has recently gained attention as a noninvasive biophysical therapy capable of enhancing nerve regeneration through mechanical stimulation and cellular activation.

**Main body:**

This review synthesizes current preclinical evidence on the effects of LIPUS across in vitro and in vivo models and outlines its underlying mechanobiological mechanisms and translational implications. A systematic search identified thirty-four experimental studies evaluating LIPUS in nerve injury models, including crush, transection, autograft, and conduit repair. Collectively, these studies demonstrate that LIPUS accelerates axonal regeneration, promotes remyelination, enhances Schwann cell proliferation and migration, and increases neurotrophic factor expression. LIPUS also facilitates neural stem cell differentiation and neuronal survival by activating key intracellular signaling pathways. Despite encouraging preclinical results, significant challenges remain in translating LIPUS to clinical practice. Variability in stimulation parameters, injury models, and species hampers reproducibility, and regulatory approval for neural applications is still pending. Future research should focus on standardizing dosimetry, validating mechanistic pathways in human tissues, and conducting multicenter clinical trials to establish safety, efficacy, and practical treatment guidelines.

**Short conclusion:**

The current evidence supports LIPUS as a safe and adaptable mechanotransductive therapy that can modulate cellular behavior and molecular signaling to enhance peripheral nerve regeneration.

**Graphical Abstract:**

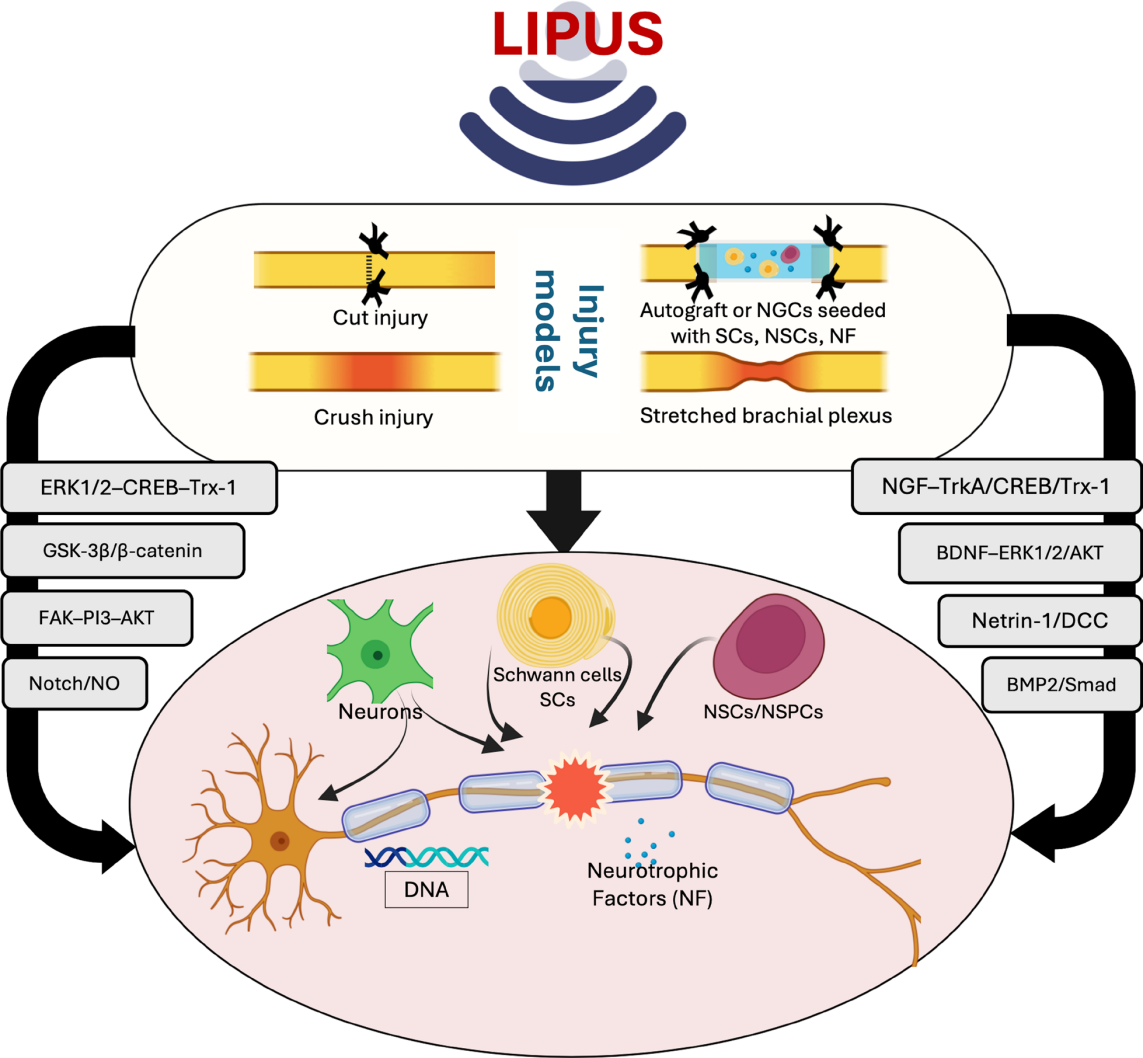

## Introduction

Peripheral nerve injury (PNI), frequently encountered in clinical practice, results in deficits in sensory, motor, and autonomic functions. Its causes range from trauma to infection and autoimmune disease [1]. Despite significant surgical advances in nerve repair and reconstruction, outcomes remain limited, particularly in cases involving severe or proximal injuries. Although the peripheral nervous system (PNS) demonstrates a greater regenerative capacity than the central nervous system (CNS), its overall ability to repair remains restricted. As a result, many patients–often young and previously healthy–face long-term disability due to incomplete or delayed functional recovery [2, 3].

PNI often results in incomplete recovery due to limited intrinsic regeneration and suboptimal outcomes with current surgical approaches, such as tension-free repairs, nerve transfers, autografts, and nerve conduits [3–10]. One promising strategy to enhance repair involves stimulating natural physiological processes, particularly the activity of Schwann cells (SCs), which play a central role in Wallerian degeneration, debris clearance, axon guidance, and neurotrophic support [11, 12]. Advances in tissue engineering and biomaterials have improved the design of nerve guidance conduits (NGCs), incorporating stem cells, growth factors, and bioactive polymers to better replicate the native regenerative environment [13–16]. Parallel efforts using gene therapy, pharmacological agents, and nanotechnology-based approaches have sought to promote axonal growth and functional recovery [17, 18]. Beyond biochemical and structural interventions, physical stimulation techniques have recently gained attention for their ability to modulate the cellular microenvironment and enhance regeneration. These include light, magnetic, and electrical stimulation, as well as ultrasound-based methods [19, 20].

While these advances mark significant progress, several critical challenges remain unresolved. Many current therapies still struggle to effectively bridge extensive nerve gaps, achieve consistent functional recovery, and replicate the complex cellular interactions required for successful regeneration [13, 21]. Additionally, their clinical adoption is limited by factors such as invasiveness, cost, and the need for specialized handling. In this context, low-intensity pulsed ultrasound (LIPUS) has gained increasing attention as a promising adjunct or alternative. LIPUS has been a clinically recognized modality for promoting bone healing for nearly three decades [22, 23]. Since then, research has expanded its potential applications across various tissues, demonstrating that LIPUS can influence cellular behavior, modulate inflammation, and stimulate regeneration. Specifically, it has been shown to accelerate the repair of soft tissues such as muscle, tendon, and ligament, as well as to modulate neural activity [24–35]. LIPUS is now being explored as a non-invasive strategy to stimulate key cellular processes involved in nerve repair [2, 36, 37]. By delivering mechanical cues, LIPUS enhances Schwann cell (SC) function, boosts the production of neurotrophic factors like brain-derived neurotrophic factor (BDNF) and nerve growth factor (NGF), improves angiogenesis, and promotes both axonal growth and remyelination. Its favorable safety profile and noninvasive nature make LIPUS an attractive option for encouraging nerve and stem cell proliferation, directing differentiation, and enhancing neurotrophic support. Numerous preclinical studies have confirmed that LIPUS facilitates peripheral nerve regeneration through diverse biological effects, including strengthening SC responses, dampening inflammation, and accelerating axonal extension [16, 38–44].

Despite these promising findings, the application of LIPUS in PNI and nerve regeneration remains relatively limited and underexplored [11]. This review aims to present the scientific rationale for using LIPUS in the treatment of PNI, summarize its reported effects across various nerve injury models, and examine the underlying cellular and molecular mechanisms, particularly its influence on SC and regenerative signaling pathways. By evaluating current preclinical evidence across different injury types (crush, stretch, neurotomy, primary repair, and graft/conduit), we seek to clarify the potential role of LIPUS in nerve regeneration and highlight key areas for future clinical application.

## Methods

Between 2001 and 2024, we systematically searched five major databases, including Ovid-Medline, Embase, PubMed, Web of Science, and Scopus, to gather preclinical research focused on the role of LIPUS in promoting peripheral nerve regeneration. An initial scoping search was conducted using Google Scholar to assess the breadth of available literature. Based on the volume of preliminary data, the search was then refined to focus exclusively on peer-reviewed sources within the selected databases.

The literature search employed a range of terms, including variations such as “*low-intensity ultrasound and nerve regeneration*”, “*low-intensity focused ultrasound*”, “*low-intensity pulsed ultrasound and nerve regeneration*”, along with other related combinations involving nerve regeneration and LIPUS stimulation. Search strategies were developed using the population, intervention, comparison, outcome (PICO) framework and adapted with syntax appropriate to each database. All retrieved records were imported into EndNote 21 for reference management, and 102 duplicate entries were removed from an initial total of 175.

The remaining 73 unique records were screened based on titles and abstracts to exclude non-relevant, non-preclinical, or non-ultrasound-related studies. Of these, 46 full-text articles were further evaluated for eligibility according to predefined inclusion criteria: (1) preclinical animal models of peripheral nerve injury; (2) use of LIPUS as an intervention; and (3) quantifiable outcomes related to nerve regeneration or functional recovery. Studies were excluded if they lacked sufficient methodological detail (e.g., missing ultrasound parameters), used non-comparable ultrasound intensities, or focused solely on carpal tunnel syndrome models. Ultimately, 34 preclinical studies met all criteria and were included in the final synthesis. This structured screening process ensured the inclusion of relevant and methodologically sound evidence to map the current landscape of LIPUS applications in peripheral nerve regeneration research.

**Fig. 1:**
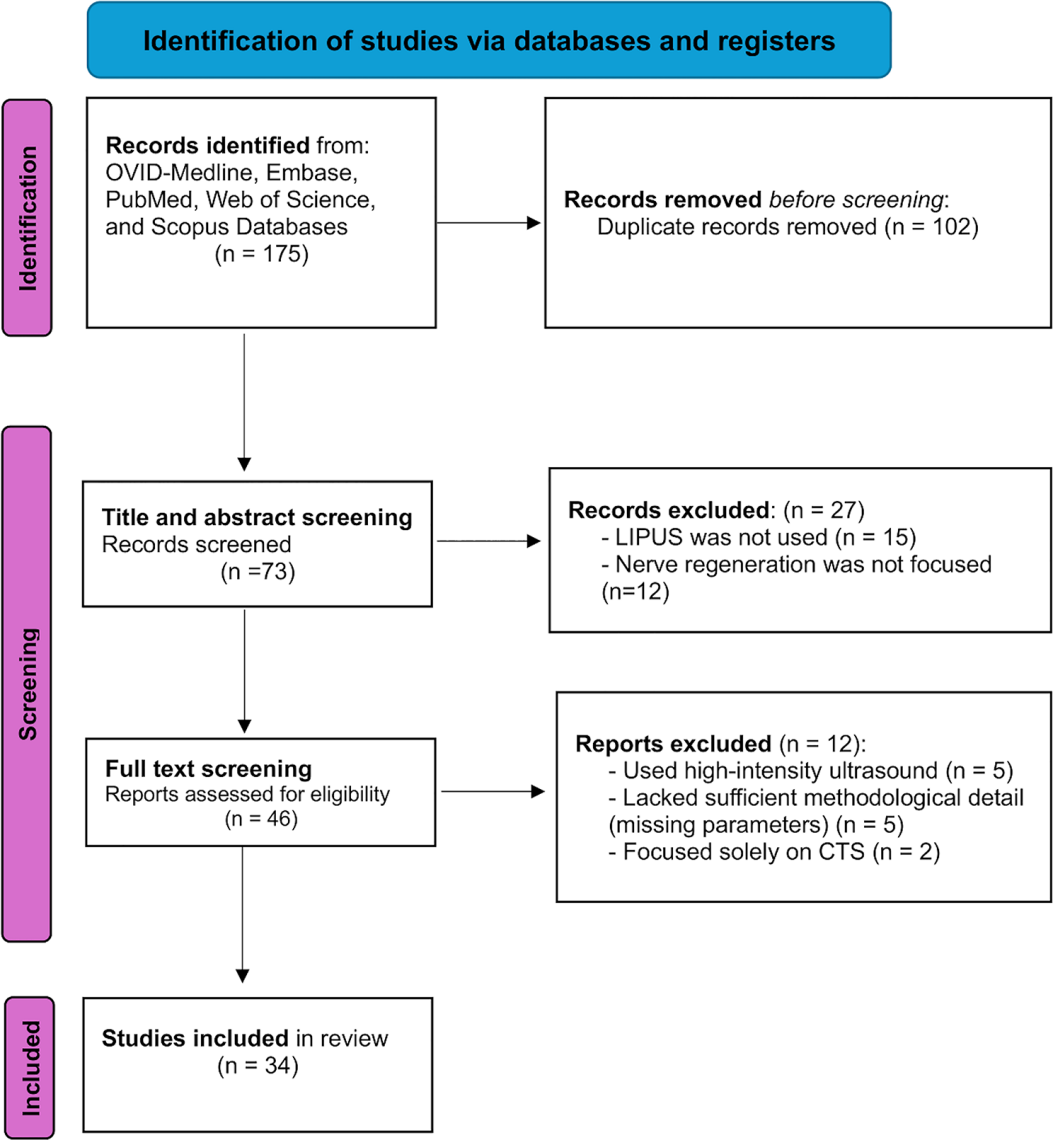
PRISMA flow diagram of study identification, screening, and inclusion for preclinical studies on LIPUS in peripheral nerve regeneration. A total of 175 records were identified, and 102 duplicates were removed. The remaining 73 records were screened by title and abstract, excluding 27 non-relevant studies. Of 46 full-text articles assessed for eligibility, 12 were excluded due to high-intensity ultrasound use, incomplete methodological data, or focus on carpal tunnel syndrome models (CTS). Ultimately, 34 preclinical studies meeting inclusion criteria were retained for qualitative synthesis. The review followed PRISMA guidelines for transparency and methodological rigor, though a formal meta-analysis was not conducted due to the heterogeneity and limited grading of preclinical evidence

This review was conducted in accordance with the preferred reporting items for systematic reviews and meta-analyses (PRISMA) guidelines to ensure transparency and comprehensive reporting. However, a full systematic review or meta-analysis was not performed due to the lack of graded levels of evidence in the included preclinical animal studies (Fig. 1).

## Mechanism of peripheral nerve regeneration after injury

Following PNI, the axon and myelin undergo degeneration and exhibit region-specific biological responses depending on their location relative to the injury site. The portion of the nerve proximal to the lesion retracts and undergoes retrograde degeneration, characterized by cell body swelling, nuclear displacement, and dissolution of Nissl bodies [45]. Distally, Wallerian degeneration begins within 24–48 hours, during which axons fragment, myelin forms ovoids, and SCs and infiltrating macrophages cooperate to clear debris. As debris removal progresses, SCs transdifferentiate into a repair phenotype and align into longitudinal Büngner bands that guide regenerating axons. Axonal elongation toward the distal stump is supported by SC-derived trophic factors and extracellular matrix cues, ultimately followed by SC redifferentiation and remyelination, which restore conduction and functional recovery. This chronological framework provides essential context for understanding subsequent cellular mechanisms and how LIPUS interacts with each phase of regeneration.

### Axonal degeneration phase

#### Axonal breakdown

Wallerian degeneration begins shortly after injury and proceeds more rapidly in thinner nerve fibers than in thicker ones. This process is initiated by an influx of calcium ions (Ca^2 +^), which activate proteases like calpain, leading to structural damage of the axon. These enzymes degrade key axonal structural components, including intermediate filaments, cellular organelles (e.g., mitochondria and elements of the endoplasmic reticulum), and the cytoskeletal framework. SCs also permit Ca^2 +^ entry, further promoting protease activation and release [46–48].

#### Myelin debris clearance

After injury, SCs undergo functional and structural changes to facilitate the repair and restoration of the damaged nerve. SCs exist in two main types: myelinating SCs, which wrap around large axons to form myelin sheaths, and non-myelinating SCs, which include Remak SCs (that ensheath small axons) and terminal SCs (located at neuromuscular junctions, where they aid in repair) [49, 50].

Upon injury, SCs become activated and release cytokines and chemokines that recruit macrophages. Together, SCs and macrophages clear cellular debris and release signals that promote axon growth. Early in Wallerian degeneration, SCs secrete monocyte chemoattractant protein-1 (MCP-1), tumor necrosis factor-alpha (TNF-α), interleukin-1 beta (IL-1β), interleukin-6 (IL-6), and leukemia inhibitory factor (LIF), all of which help attract macrophages to the injury site. This pro-regenerative environment supports the formation of Büngner bands–longitudinal SC structures that guide regenerating axons [51, 52].

**Fig. 2:**
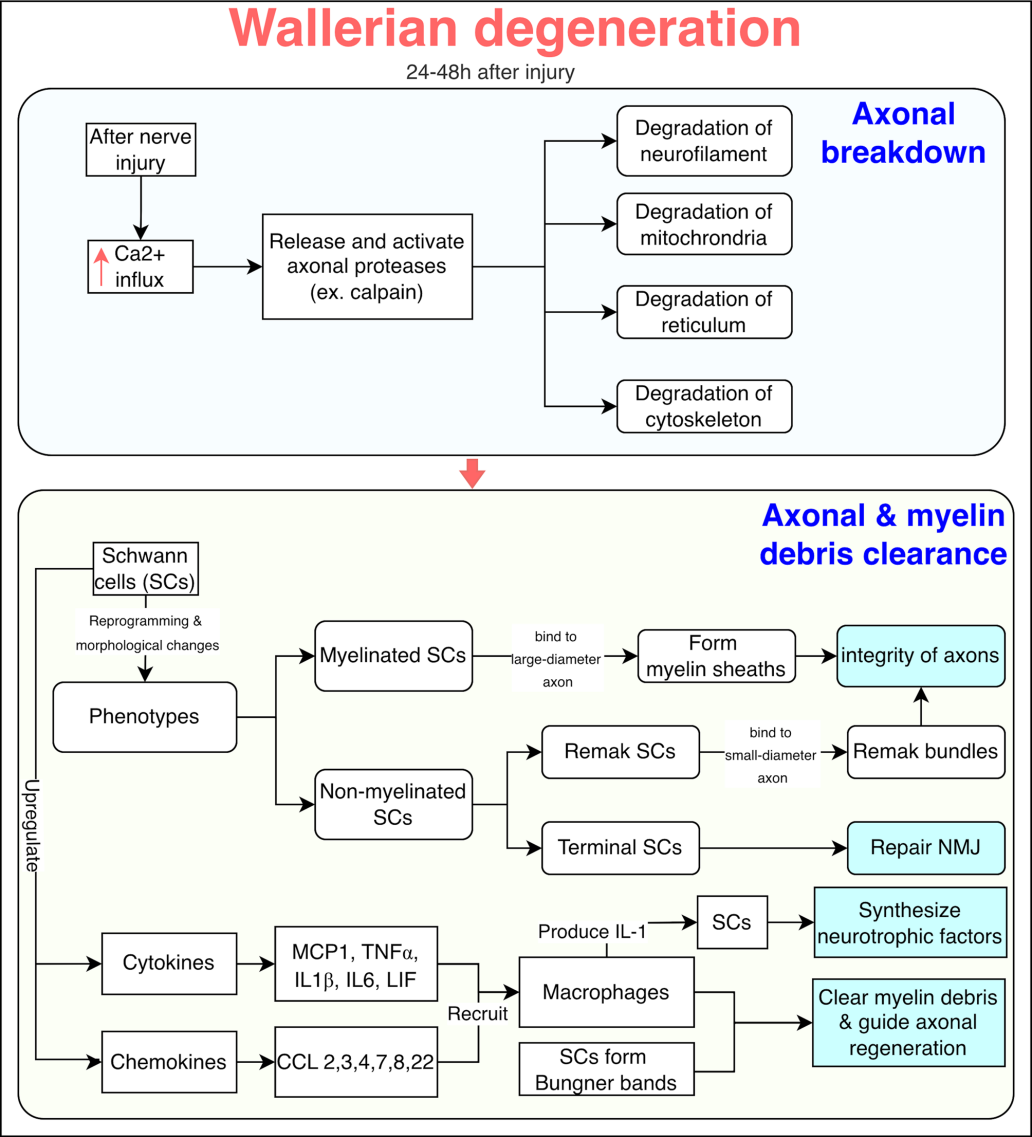
Wallerian degeneration process showing axonal breakdown and myelin debris clearance phases. Axonal breakdown is triggered by Ca^2 +^ influx, activating calpain proteases and leading to degradation of neurofilaments, mitochondria, reticulum, and cytoskeleton. In the clearance phase, SCs undergo phenotypic changes, release cytokines and chemokines to recruit macrophages, and support debris removal and axonal regeneration through neurotrophic signaling. Cytokines also promote SCs to form the Büngner bands that guides sorting axon, while IL-1 secreted by macrophages induce SCs secrete neurotrophic factors. SCs: schwann cells; NMJ: neuromuscular junction; MCP1: monocyte chemoattractant protein-1; TNFα: tumor necrosis factor-alpha; IL1β: interleukin-1 beta; IL6: interleukin-6; LIF: leukemia inhibitory factor; CCL: C-C motif chemokine ligand

SC-macrophage interactions also regulate inflammation and facilitate debris clearance, further supporting axonal branching. Recent findings show IL-17B/IL-17RB signaling in SCs boosts chemokine expression (e.g., C-C motif chemokine ligand (CCL) 2, CCL3, CCL4, CCL7, CCL22, CCL8), promoting additional macrophage recruitment. Moreover, IL-1 produced by macrophages stimulates SCs to secrete neurotrophic factors such as NGF, further contributing to the regenerative process [53, 54] (Fig. 2).

#### Axonal regeneration phase

Following debris clearance, repair SCs undergo proliferation and elongation, reorganizing into longitudinally aligned cords known as Büngner bands [51, 52]. These structures define the path along which regenerating axons extend toward their distal targets. SCs secrete neurotrophic factors, such as NGF, BDNF, NT-3, and GDNF, that promote axonal elongation, cytoskeletal remodeling, and growth cone guidance [49, 55]. Concurrently, fibroblasts and endothelial cells contribute to extracellular matrix remodeling and angiogenesis, further supporting the regenerative milieu. As newly sprouted axons navigate through the endoneurial tubes, their progress is influenced by the balance of permissive cues (e.g., neurotrophins, laminin, cell adhesion molecules) and inhibitory signals (e.g., cytokines, axon-repelling factors). Successful regeneration depends on timely SC support, aligned guidance structures, and an environment that minimizes chronic inflammation or fibrosis [56].

#### Remyelination phase

Once regenerating axons reach their target tissues, SCs begin withdrawing from the repair phenotype and reinitiate their myelinating program [56]. These SCs realign along the regenerated axons, extend their membranes to form compact myelin, and restore saltatory conduction. This transition involves re-expression of key myelin genes and transcription factors, including Krox20, myelin protein zero (P0), and myelin basic protein (MBP), that drive the maturation of myelinating SCs [57–59]. Proper remyelination is crucial for regaining nerve conduction velocity, synaptic reinnervation, and ultimately, restoring motor and sensory function. Disturbances in this phase, such as misdirection of regenerating axons or inadequate SC differentiation, can limit the completeness of recovery.

#### Nerve regeneration – integrated signaling pathways

Peripheral nerve regeneration is governed by a coordinated network of signaling pathways that support axonal growth, SCs activation, and remyelination. These processes rely on a stable extracellular matrix (ECM), where the basal lamina forms a scaffold for SCs to align into Büngner bands and guide regenerating axons [60, 61].

Following injury, axoplasmic Ca^2 +^ influx triggers the activation of adenylate cyclase, leading to increased cyclic adenosine monophosphate (cAMP) production [62, 63]. Stabilized by neurotrophic factors such as BDNF, cAMP activates the Protein kinase A (PKA)- cAMP-regulated enhancer B (CREB) signaling pathway, enhancing the transcription of key neuroregenerative genes, including growth-associated protein 43 (GAP-43), actin, and tubulin, which are essential for cytoskeletal remodeling and growth cone development [64–66].

In parallel, Ca^2 +^ activates protein kinase C (PKC), which stimulates the Raf/Mitogen-activated protein kinase/ERK kinase (MEK)/Extracellular signal-regulated protein kinase (ERK) or Raf/MEK/Mitogen-activated protein kinase (MAPK), and phosphoinositide 3-kinase (PI3K)/protein kinase B (Akt) pathways, thereby promoting SC survival and reducing apoptosis [67]. Neurotrophic factors play a central role: NGF activates the Tropomyosin receptor kinase (TrkA)–ERK1/2–CREB signaling axis to promote neurite outgrowth [55], while BDNF binds to (Tropomyosin receptor kinase B) TrkB and initiates Ras/Raf/MEK and p38 MAPK signaling to support SCs dedifferentiation, axonal growth, and myelination [68–70]. Additionally, BDNF activates the Janus kinases/Signal transducers and activators of transcription (JAK/STAT) signaling pathway in SCs, leading to increased cytokine secretion and further facilitating nerve regeneration [70].

**Fig. 3:**
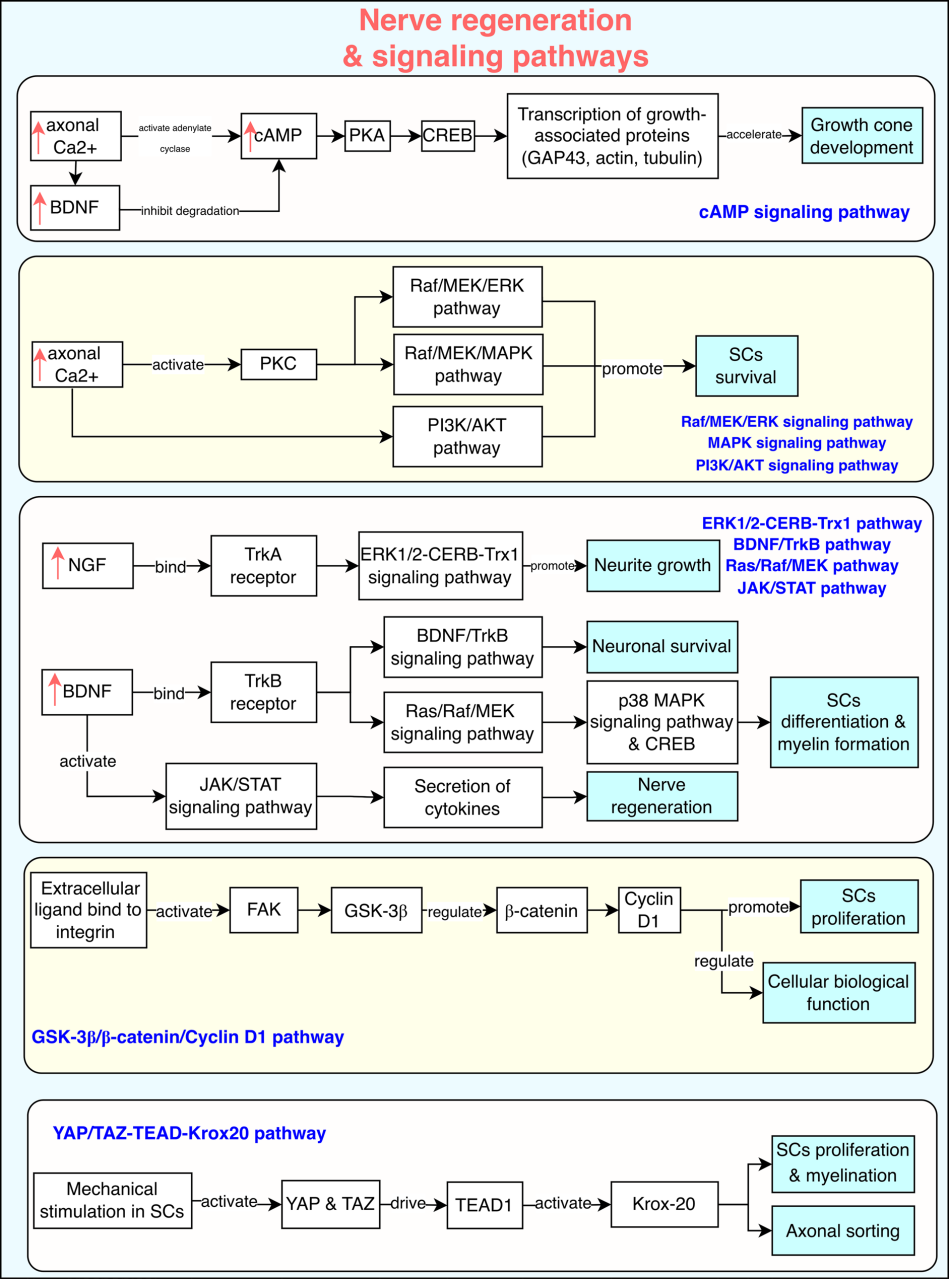
Cellular signaling pathways involved in nerve regeneration following peripheral nerve injury. Key pathways, including cAMP, Raf/MEK/ERK, PI3K/Akt, and JAK/STAT, support axonal growth as well as SC survival, differentiation, and myelination. Neurotrophic factors such as BDNF and NGF activate Trk receptors, leading to neurite outgrowth and cytokine release. Additional signaling pathways, such as GSK3β/β catenin and YAP with TAZ, regulate sc proliferation, function, and axon sorting. cAMP: cyclic adenosine monophosphate; PKA: protein kinase A; GAP-43: growth- associated protein-43; CREB: cAMP-regulated enhancer B; BDNF: brain-derived neurotrophic factor; PKC: protein kinase C; Akt: protein kinase B; MEK: mitogen-activated protein kinase/ERK kinase; ERK: extracellular signal-regulated protein kinase; NGF: nerve growth factor; Trk-A, Trk-B: tropomyosin receptor kinase A, B; JAK: Janus kinases; STAT: signal transducers and activators of transcription; PI3K: phosphatidylinositol-3-kinase; mTOR: mechanistic target of rapamycin; Trx-1: thioredoxin-1; FAK: focal adhesion kinase; GSK3β: glycogen synthase kinase 3 beta; NT-3: neurotrophin-3; SCs: schwann cells; YAP: yes associated protein; TAZ: transcriptional co-activator with PDZ-binding motif; TEAD: tea domain transcription factor

The integrin/Focal adhesion kinase (FAK) signaling cascade also contributes by activating glycogen synthase kinase 3 beta (GSK-3β)/β-catenin and cyclin D1, thus regulating SC proliferation, cellular biological function and axonal regeneration through β-catenin-mediated transcriptional control [12, 63]. Furthermore, the yes associated protein/Transcriptional co-activator with PDZ-binding motif- TEA domain transcription factor 1 (YAP/TAZ–TEAD1)–Krox20 signaling pathway–responsive to mechanical cues–plays a key role in regulating SCs proliferation, SC-mediated myelination and axonal sorting [57–59] (Fig. 3).

## Physical characteristics of ultrasound and low-power pulsed therapeutic ultrasound

Ultrasound refers to acoustic waves with frequencies above 20 kilohertz (kHz), exceeding the range of human hearing, and is widely applied in both diagnostic and therapeutic medicine. The physical characteristics of ultrasound include its high frequency, which allows for variable tissue penetration and resolution, and its propagation as mechanical waves through biological tissues. The interaction of ultrasound with tissues depends on properties such as frequency, wavelength, velocity, and acoustic impedance, which together determine how ultrasound is reflected, absorbed, or transmitted within the body [71–75]. Within the spectrum of therapeutic ultrasound, low-dose ultrasound has gained attention for its ability to induce biological effects without significant tissue heating. It typically operates at frequencies ranging from 1 to 3 MHz and at intensities below 1 watt per square centimeter (1 W/cm^2^), making it suitable for non-destructive therapeutic interventions [11, 33, 76].

LIPUS is a distinct form of low-power acoustic stimulation that delivers ultrasound energy in pulsed waves at even lower intensities–commonly around 30–200 mW/cm^2^ –and at frequencies of 0.5–1.5 MHz [77]. The pulsed nature of LIPUS minimizes thermal effects while maintaining mechanical stimulation of tissues. LIPUS has gained recognition as a non-intrusive method for therapeutic intervention, particularly in traumatology and regenerative medicine [11, 78]. Its efficacy is well established in promoting bone fracture healing, accelerating soft tissue regeneration, and modulating inflammatory responses [24, 27, 79, 80]. The biological effects of LIPUS are believed to be mediated by mechanical stimulation at the cellular level, influencing molecular pathways that regulate cellular proliferation, differentiation, and tissue repair [32, 81, 82]. LIPUS is recognized for its unique properties as a safe, and non-invasive therapy with demonstrated benefits in tissue healing and regeneration. Ongoing research continues to elucidate its mechanisms and expand its clinical applications.

## Preclinical evidence of LIPUS in promoting peripheral nerve regeneration

### In vitro studies

**Fig. 4:**
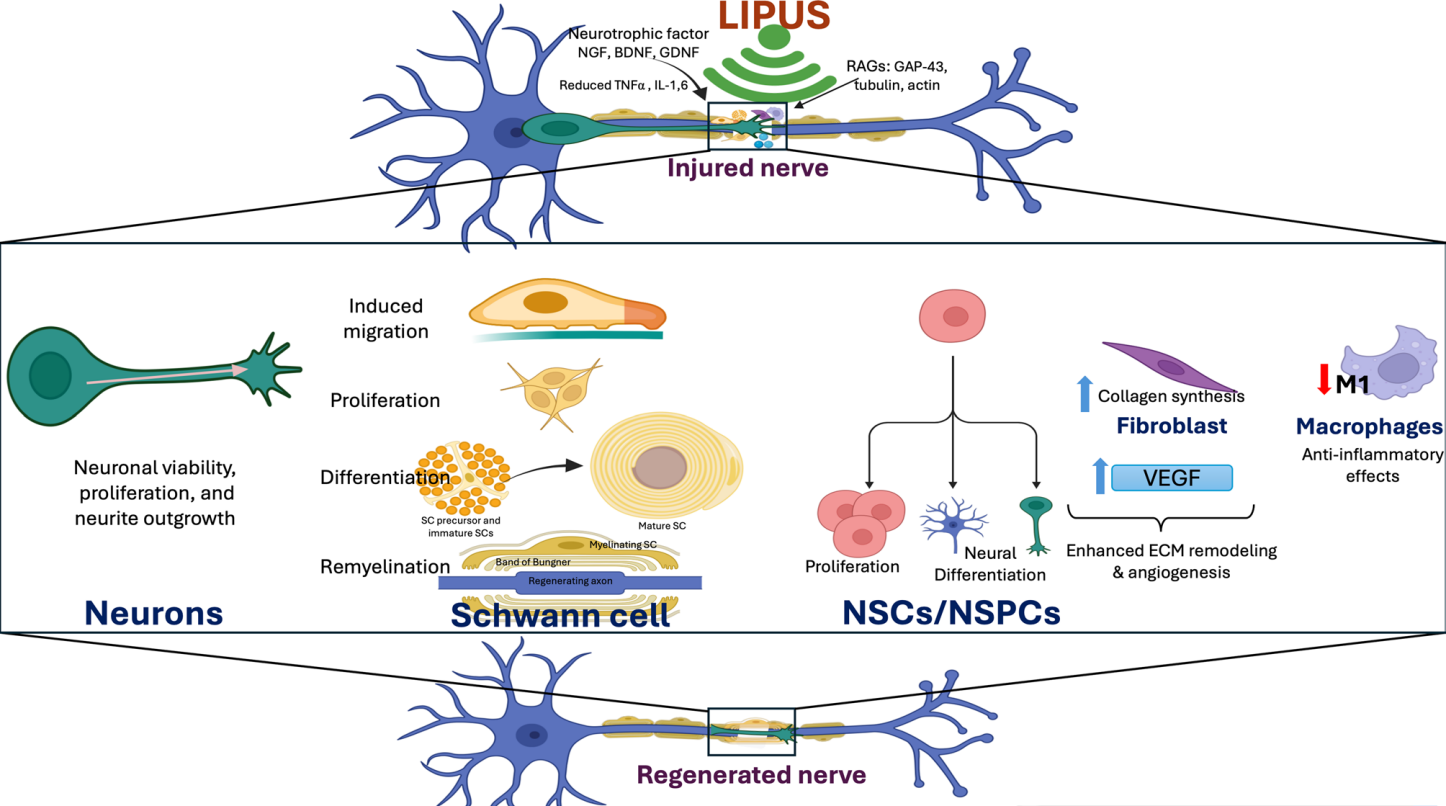
Illustration of the role of LIPUS in promoting peripheral nerve regeneration based on previous preclinical studies, particularly in vitro investigations. The effects of LIPUS are categorized into three primary cellular targets. First, LIPUS activates neurons, stimulating axonal growth toward the distal stump and facilitating injury bridging. Second, it enhances the activity of schwann cells, the key glial cells in nerve repair, by promoting their proliferation, differentiation, and remyelination, thereby accelerating nerve repair. Third, LIPUS stimulates neural stem/progenitor cells (NSCs/NSPCs) to differentiate into neuronal and glial lineages, supporting neurite outgrowth and contributing to the overall regenerative process. Moreover, LIPUS stimulates fibroblasts and endothelial cells to promote ECM remodeling and fosters an anti-inflammatory environment for regeneration by reducing pro-inflammatory M1 macrophage activity

Extensive preclinical in vitro studies have established LIPUS as a promising non-invasive approach for promoting nerve regeneration by activating multiple target cell types and engaging diverse cellular and molecular pathways (Table 1) (Fig. 4).

**Table 1:** Regenerative effects of low-intensity pulsed ultrasound in vitro studies. This table summarizes findings from cell-based studies evaluating the impact of low-intensity pulsed ultrasound (LIPUS) on neural and glial cell populations, including schwann cells, neurons, and neural stem/progenitor cells. Outcomes include proliferation, survival, differentiation, migration, and molecular signaling changes

Target cell	Parameter	Protocol	Effects	Mechanism	Reference
SCs	300 mW/cm^2^, 1.0 MHz	3 min/d	- The regenerative effects of LIPUS are likely mediated through interactions with the transplanted Schwann cells, particularly within biodegradable PLGA conduits - PLGA conduits exposed to ultrasound stimulation exhibited decreased lactate dehydrogenase (LDH) release and elevated MTT assay values, indicating reduced cytotoxicity and enhanced cell viability	LIPUS may modulate the function of membrane-bound calcium and proton ATPases, along with other channel proteins critical for maintaining cellular viability and supporting proliferative activity	Chang, 2005 [15]
SCs	300 mW/cm^2^, 1.0 MHz	3 min/d, 2d	LIPUS enhanced SCs expansion and inhibited apoptosis	LIPUS may also regulate the expression of crucial genes involved in neural repair, such as pro-inflammatory cytokines (*IL-1β*, *TNF-α*), myelin-associated proteins (*MPZ-P0*), and morphogenic factors like *Desert Hedgehog* (*Dhh*), which collectively contribute to inflammation control, myelination, and nerve regeneration	Tsuang, 2011 [41]
SCs	27.37 mW/cm^2^, 1.0 MHz	10 min/day, 5d	LIPUS improved Schwann cell survival and growth, aiding nerve repair	- Activation of the glycogen synthase kinase-3 beta (GSK-3β) pathway leading to β-catenin stabilization and increased Cyclin D1 expression, which supports cell cycle progression - Upregulation of growth-promoting proteins such as BDNF, GDNF, FGF, and NGF	Ren, 2018 [12]
SCs	- MPG/CN explant: 0, 50, 100, 200, 300, and 500 mW/cm^2^, 1.0 MHz - SCs: 80 mW/cm^2^, 1,0 MHz	10 min/d, daily, 3 sessions at 2 h, 24 h, 48 h after seeding	- The study investigated LIPUSs effects on Schwann cells to promote nerve regeneration and treat erectile dysfunction (ED) - LIPUS-SCs-Exo enhanced CN regeneration and restores erectile function in BCNI rats	- Identified the PI3K-Akt-FoxO signaling pathway, promoting neurite outgrowth	Kun-Ye, 2023 [83]
SCs	-	-	Both therapies enhanced Schwann cell growth, suppressed inflammation, and created a more favorable environment for regeneration	- Neurotrophic factors: NGF and BDNF for neuronal viability, myelin sheath restoration, and adaptability of synaptic connections - Cytokines: TGF-β and IL-10 in modulating inflammation and promoting axonal regeneration	Siwak, 2024 [84]
NSCs	69.3 mW/cm^2^, 1.0 MHz	5 min/day, 3d	Neural stem cell growth and specialization were enhanced by LIPUS under in vitro conditions	- The Notch signaling pathway, involving receptors like Notch1 and effector Hes1 - Upregulation of neural growth-supporting proteins, including nerve growth factor (NGF), neurotrophin-3 (NT-3), and brain-derived neurotrophic factor (BDNF)	Wu, 2020 [85]
NSCs	25 or 50 mW/cm^2^, 1.5 MHZ	10 min/d, 4d	LIPUS may enhance neurite outgrowth, potentially aiding neurodegenerative disease therapies	- mTOR, ERK1/2, and Protein kinase B (Akt) as key signaling pathways - BDNF is identified as a significant neurotrophic factor	Xuanjie-Ye, 2023 [86]
NSCs	60 mW/cm^2^, 1.5 MHz	20 min/session, 1 or 3 session/d, 3d	LIPUS markedly promoted the proliferation of neural stem cells derived from the SH-SY5Y cell line	- BDNF is known to support neuronal survival and growth - Potential signaling pathways implicated in this process include the ERK signaling pathway	Arwa A. Al-Maswary, 2024 [87]
iPSCs–NCSCs	100, 300, 500, 700, 900, 1100, 1300, 1500 mW/cm^2^, 1.0 MHz	10 min/d, 2d − 4d	LIPUS enhanced cell survival, boosted growth, and promoted neural specialization of iPSCs–NCSCs.	The expression levels of NF-M, Tuj1, S100b, and GFAP genes and their corresponding proteins were elevated in iPSC-derived neural crest stem cells (iPSCs–NCSCs)	Yonggang, 2013 [88]
NSPCs	100, 500 mW/cm^2^, 1.8 MHz	5 min/session, a 3d interval between sessions, 5-11d	LIUS facilitated maturation and stimulated neurite extension in neural stem/progenitor cells (NSPCs)	- Nitric oxide (NO) Is a key contributor to the cellular mechanisms of neural multipotent neural progenitors(NSPCs) - Neurotrophins - for example NGF, are highlighted as significant enhancers of neuronal differentiation	Lee, 2014 [43]
NSPCs	533 mW/cm^2^, 1138 and 560 KHz	5 min/sesion, 2–3 sesion, 2d	LIUS promoted undifferentiated neural cell (NSPC) specialization and attachment	Activate mechanosensitive channels on the cell membrane, leading to increased intracellular calcium levels	Lee, 2019 [42]
PC12 cells	30 or 50 mW/cm^2^, 1.0 MHz	10 min/d, every other day, 7d	The synergy of LIPUS and NGF, applied at their specified intensity and dosage, substantially promoted neural projection growth beyond what is achieved with NGF by itself	- NGF binds TrkA, activating (MEK)/(ERK) and (PI3K)/Akt pathways - The ERK1/2-mediated pathway leading to CREB activation and downstream Trx-1 expression	Lu Zhao, 2016 [55]
Sensory neuron	0.5 MHz, 200–800 mV	3 min	Application of ultrasound to cultured dorsal root ganglion (DRG) neurons altered neurite structure and promoted overall neurite elongation.	-	Ventre 2018 [89]
Cortical neuronal cell/primary culture	0, 8, 120, and 210 mW/cm^2^, 1.0 MHz	5 min/d, 10d	Promotes axonal regeneration and inhibits neuronal apoptosis	Activating netrin-1 and DCC expression	Wen, 2021 [90]

### Schwann cell target

A growing body of preclinical evidence identifies SCs—the principal glial cells of the peripheral nervous system—as a key cellular target through which LIPUS exerts its regenerative effects in PNI. SCs are essential for axonal maintenance, myelin formation, and the orchestration of a pro-regenerative microenvironment following injury. Numerous in vitro studies, including those by Chang et al. [15], Tsuang et al. [41], Ren et al. [12], and Siwak et al. [84], demonstrate that LIPUS significantly enhances SC viability, proliferation, and migration—even under adverse conditions such as serum deprivation. These effects are accompanied by reduced apoptotic activity and a shift in gene expression toward a regenerative phenotype.

LIPUS also modulates key molecular pathways in SCs. For example, Ren et al. [12] showed that LIPUS activates the GSK-3β/β-catenin/Cyclin D1 axis, promoting SC proliferation and differentiation. This is further supported by increased expression of neurotrophic factors, including NGF, BDNF, glial cell line-derived neurotrophic factor (GDNF), and fibroblast growth factor (FGF), which collectively enhance SC function and support neuronal survival and axon outgrowth. Concurrently, LIPUS suppresses pro-inflammatory mediators such as IL-1 and TNF-α [41], while upregulating anti-inflammatory and regenerative markers such as IL-10 [84] and Desert Hedgehog (Dhh) [41], thus contributing to a more favorable microenvironment for nerve repair.

In addition to promoting SC proliferation and survival, LIPUS preserves SC identity by upregulating critical myelin-associated genes such as P0 and Krox-20 [41], both essential for myelination. Crisci et al. [39] and Siwak et al. [84] further reported enhanced SC migration and activity, which correlates with accelerated axonal regeneration and increased myelinated fibers. These in vitro findings are supported by in vivo studies. Research by Wang et al. [91], Sato et al. [92], and Raso et al. [93] demonstrated that LIPUS promotes neurotrophin-3 (NT3) release, supports the restoration of neural tube architecture, and facilitates remyelination. Importantly, the regenerative benefits of LIPUS in scaffold-based therapies—such as autografts and Poly(lactic-co-glycolic acid) (PLGA) conduits—appear to depend significantly on its capacity to support the implanted SC population, as noted by Jiang et al. [94] and Chang et al. [95].

Collectively, these findings underscore the central role of SCs in LIPUS-mediated nerve repair and highlight the therapeutic potential of targeting SC biology to enhance therapeutic outcomes in peripheral nerve regeneration.

### Neuronal target

While SCs are central to peripheral nerve regeneration, mounting evidence indicates that LIPUS also directly promotes neuronal repair by stimulating neurite outgrowth, enhancing axonal regeneration, and supporting neuronal survival. In vitro studies using PC12 cells, dorsal root ganglia (DRG), primary cortical neurons, and neuroblastoma cells consistently demonstrate that LIPUS enhances neurite extension, increases secondary branching, and promotes axonal elongation [55, 83, 89, 90]. These structural changes are associated with the modulation of several key intracellular signaling pathways.

LIPUS synergizes with neurotrophic factors such as NGF to activate the ERK1/2 signaling cascade, which subsequently stimulates CREB and upregulates thioredoxin-1 (Trx-1), a protein critical for neuronal survival and differentiation. Additionally, LIPUS activates the netrin-1/deleted in colorectal carcinoma (DCC) pathway—important in axon guidance—and influences the mechanistic target of rapamycin (mTOR) and PI3K/Akt/forkhead box O (FoxO) signaling axes, which regulates cellular growth, metabolism, and resistance to oxidative stress. Deactivation of GSK-3β, another effect of LIPUS, further contributes to enhanced axonal outgrowth [89, 92].

Beyond structural support, LIPUS exhibits strong neuroprotective properties. Wen et al. [90] reported significant increases in neuronal survival and reductions in programmed cell death following LIPUS treatment. Chen et al. [96] and Ni et al. [97] showed that LIPUS upregulates neurotrophic factors such as NGF and BDNF, both at the injury site and in DRG neurons. Similarly, Ren et al. [12] found that LIPUS stimulates a broader spectrum of neurotrophic factors—including FGF, NGF, BDNF, and GDNF—all of which are critical for neuronal differentiation, axonal growth, synaptic plasticity, and long-distance connectivity. In PC12 and SK-N-SH neuroblastoma models, Lu Zhao et al. [55], Ye et al. [86], and Siwak et al. [84] demonstrated that LIPUS promotes neurite elongation and differentiation, particularly when combined with NGF, suggesting a synergistic mechanism.

Together, these findings underscore the capacity of LIPUS to directly modulate neuronal function and structure, making it a multifaceted tool for enhancing both cellular survival and the regenerative architecture required for functional nerve repair.

### Neural stem cells and other cellular targets of LIPUS

LIPUS has emerged as a promising noninvasive strategy to promote neural regeneration by directly targeting multipotent stem cell populations such as neural stem/progenitor cells (NSPCs) and neural crest stem cells derived from induced pluripotent stem cells (iPSC-NCSCs). These cells possess the capacity to proliferate, differentiate, and contribute to neural repair following injury. Numerous studies have shown that LIPUS significantly enhances their survival, proliferation, and differentiation into neuronal and glial lineages.

Yonggang Lv et al. [88, 98] and Xia et al. [99] demonstrated that LIPUS exposure enhances the viability, mitotic activity, and neurogenic potential of iPSC-NCSCs, accompanied by upregulated expression of neuronal and glial markers such as neurofilament medium chain (NF-M), class III β-tubulin (Tuj1), S100β, and glial fibrillary acidic protein (GFAP). These effects are mediated by activation of the FAK–ERK1/2 signaling cascade, a key regulator of stem cell fate and neural outgrowth. Similarly, Lee et al. [42, 43] and Wu et al. [85] reported that LIPUS promoted neurite extension, neuronal network formation, astrocytic differentiation and improved cell attachment in NSPCs. Notably, dual-frequency ultrasound was found to enhance these outcomes more effectively than single-frequency stimulation.

Further mechanistic insights revealed that nitric oxide and neurotrophic factors such as NGF mediate many of these responses. Wu et al. [85], Ye et al. [86], and Al-Maswary et al. [87] confirmed that LIPUS promotes neural stem cell proliferation and lineage specification through modulation of signaling pathways including Notch, mTOR, ERK1/2, and Akt. These changes were accompanied by elevated levels of neurotrophic factors such as NGF, NT-3, and BDNF, supporting both cell survival and synaptic integration. Notably, LIPUS stimulation of SH-SY5Y-derived neural stem cells led to significant upregulation of BDNF and activation of ERK, highlighting its targeted action without increasing apoptosis.

Beyond neural and glial populations, LIPUS also modulates additional cell types integral to the regenerative microenvironment. Raso et al. [93] observed enhanced nerve sprouting and increased fiber density, suggesting direct effects on nerve fibers and associated SCs. Sato et al. [92] reported increased collagen synthesis in fibroblasts and upregulation of vascular endothelial growth factor (VEGF) in endothelial cells, facilitating extracellular matrix remodeling and angiogenesis. Furthermore, Kawai et al. [100] demonstrated that LIPUS reduces M1 macrophage populations post-injury, indicating anti-inflammatory effects that reshape the immune microenvironment to favor regeneration.

Collectively, these findings demonstrate that LIPUS acts on a wide range of cell types, including stem cells, neurons, glia, fibroblasts, endothelial cells, and immune cells, creating a multifaceted pro-regenerative environment. Its ability to support both proliferation and directed differentiation in stem cell-derived neural models underscores its potential in future cell-based therapies aimed at functional neural restoration.

### In vivo studies – with incomplete injury models

Preclinical in vivo studies using peripheral nerve crush models have consistently demonstrated that LIPUS effectively promotes nerve regeneration through structural, functional, and molecular enhancements (Table 2). Multiple studies, including those by Mourad et al. [102] and Raso et al. [93], showed that LIPUS accelerates functional motor recovery. Treated animals exhibited earlier improvements in the sciatic functional index (SFI) and gait, with recovery beginning several days earlier than in control groups. These effects were attributed to enhanced axonal regrowth, early myelin repair, and increased vascularization and immune activity at the injury site.

**Table 2:** Morphological and functional outcome of low-intensity pulsed ultrasound in vivo studies using incomplete injury models. This table presents animal studies employing non-transection peripheral nerve injury models (e.g., crush, stretch)

Species	Model-Nerve	Parameters	Protocol	Follow-up	Outcome	Reference
NMRI mice	Crush-sciatic nerve	200, 500, 1000 mW/cm^2^; 1.0 and 3.0 MHz	2 min/day, daily, maximum 14d	14d	Altered ultrasound parameters led to more favorable outcomes compared to previous studies	Akhlagi, 2012 [101]
Lewis rat	Crush-sciatic nerve	0, 30, 140, 250 mW/cm^2^, 1.0 MHz	5 min/d, 5d/w, 1-3w	21d	- LIPUS promoted both axonal sprouting and remyelination, with the most pronounced effects observed at an intensity of 140 mW/cm^2^ - Analysis of mRNA levels indicated that ultrasound exposure downregulated the expression of pro-inflammatory proteins	Ito, 2020 [44]
Lewis rat	Crush-sciatic nerve	140 mW/cm^2^, 1.0 MHz,	5 min/d, daily in 14d and 5d/week, until death (30 days)	30d	- LIPUS significantly enhanced regenerative axonal length and myelinated nerve fiber characteristics - LIPUS treatment increased both the gene and protein expression of brain-derived neurotrophic factor (BDNF).	Wang, 2021 [91]
Lewis rat	Crush-sciatic nerve	250 mW/cm^2^, 2.25 MHz	1 min/session, 3d/week, 30d	30d	Accelerated foot function recovery from axonotmetic injury in an animal model	Mourad, 2001 [102]
Wistar rat	Crush-sciatic nerve	400 mW/cm^2^, 1.0 MHz	2 min/d, 10d	21d	- Improved SFI significantly more in treated nerves compared to untreated ones - Histologic analysis showed higher nerve fiber density in treated nerves, indicating enhanced regeneration	Raso, 2004 [93]
Wistar rat	Crush-sciatic nerve	250 mW/cm^2^, 1.0 MHz	1 min/d, every other day, 8w	56d	- LIPUS stimulated rapid nerve repair and improved functional outcomes - Improved nerve fiber density, (SFI), and sensory nerve conduction velocity (SNCV)	Chen, 2010 [96]
SD rat	Crush-sciatic nerve	200 mW/cm^2^, 1.0 MHz,	1 min/d, every other day	28d	Resulted in improved sciatic nerve function and muscle weight ratios compared to controls, by potentially stimulating BDNF release, crucial for nerve regeneration	Ni, 2016 [97]
SD rat	Stretch-Brachial plexus (BPI)	100 mW/cm^2^, 1.0 MHz	20 min/d, 12d	28d	- Combined treatment with LIPUS and methylcobalamin showed synergistic effects, enhancing functional recovery - Early intervention with these treatments reduced neuroinflammation and spinal microglia overactivation - LIPUS and methylcobalamin can modulate BPI-induced dysfunction effectively	Y. L. Hsieh, 2023 [103]
SD rat	Crush-sciatic nerve	500 mW/cm^2^, 1.1 MHz	10 min/session, 3 sessions/w, 4w	56d	- Showed improved SFI, muscle fiber diameter, and myelinated axons in the remote LIPUS group - Plasma levels of total antioxidant capacity, malondialdehyde, interleukin-6, and HSP70 differed significantly between groups	Mahsa Nosratiyan, 2024 [104]

It highlights LIPUS effects on axonal regeneration, myelination, inflammation, and functional recovery, along with key stimulation parameters and animal species used

Additional structural improvements following LIPUS application were reported in studies conducted in 2010 [96], 2016 [97], and 2021 [91], further supporting its regenerative efficacy. LIPUS treatment resulted in increased densities of myelinated nerve fibers – the number of myelinated axons per unit area within a nerve cross-section, thicker myelin sheaths – the measurable width of the myelin sheath surrounding a regenerated axon, and better-organized axonal morphology – spatial arrangement and structural alignment of regenerated axons and supporting cells within a nerve fascicle. Notably, these histological enhancements were associated with improved nerve conduction velocities and reduced signs of degeneration [91, 96, 97]. In addition, Ni and Wang found that LIPUS significantly elevated BDNF expression in both the injured nerve and DRG, thereby supporting axonal survival and myelination [91, 97].

Mechanistically, LIPUS promotes regeneration through upregulation of neurotrophic factors such as NGF and BDNF, stimulation of SC activity, and modulation of inflammatory responses. Chen et al. [96] linked increased NGF levels to improved SC function, while Ito et al. [44] demonstrated that LIPUS suppressed immune signaling molecules including TNF-α and IL-6, as well as axon-repelling factors such as semaphorin 3A (SEMA3A) and glycogen synthase kinase 3 beta (GSK3β), thereby creating a more favorable regenerative microenvironment. These molecular shifts directly support axon elongation, remyelination, and tissue repair. Akhlagi et al. [101] highlighted the importance of treatment parameters by testing 20 different LIPUS protocols. The study revealed that non-thermal, pulsed mode yielded the better outcomes, achieving up to 90% functional recovery by day 14, underscoring the critical role of mechanical effects. Two recent studies expand the therapeutic scope of LIPUS in novel and complex injury contexts. Hsieh et al. [103] investigated brachial plexus injury (BPI), a severe and challenging condition, and found that LIPUS combined with methylcobalamin significantly enhanced functional recovery by reducing neuroinflammation and modulating spinal glial activity. In contrast, Nosratiyan et al. [104] introduced the novel concept of “remote” LIPUS, wherein ultrasound was applied to the limb contralateral to the sciatic nerve injury. Remarkably, this indirect stimulation still promoted nerve regeneration by enhancing SC activity and BDNF expression, suggesting a systemic or network-based neuromodulatory effect. Collectively, these findings position LIPUS as a promising noninvasive modality with robust and innovative therapeutic potential for peripheral nerve repair, even in complex injury models.

### In vivo studies – with cut and grafted nerve models

Several in vivo studies consistently demonstrate that LIPUS significantly accelerates peripheral nerve regeneration by enhancing axonal growth and improving morphological recovery (Table 3). For instance, in a sciatic nerve neurotomy model, Crisci et al. [39] observed rapid morphological regeneration after just 12 days of daily LIPUS treatment. Similarly, Jiang et al. [94] reported elevated expression of the axonal marker neurofilament 200 (NF200), confirming a faster regeneration rate in LIPUS-treated autograft groups at 8 weeks post-injury. In conduit-based models, Park et al. [16] found that LIPUS increased the nerve regeneration rate from 0.48 mm/day to 0.71 mm/day when used with PLGA/Pluronic F127 tubes. Likewise, Kim et al. [106] showed that LIPUS not only enhanced nerve regeneration in NGCs but also outperformed NGF stimulation alone. Beyond accelerating axonal growth, LIPUS has been shown to improve the structural integrity of regenerating nerves. Crisci et al. [39] noted an increase in fiber types within mixed nerves after LIPUS treatment, suggesting improved structural recovery. This trend continued in studies using PLGA conduits, where LIPUS led to a greater number and larger cross-sectional area of newly formed nerve fibers, particularly at the midpoint of the conduit [15, 95]. Notably, the inclusion of SCs within the conduits further amplified the regenerative effects of LIPUS, indicating a synergistic interaction. However, conduit material significantly influenced outcomes: silicone conduits were associated with poor regeneration due to fibrous tissue formation that impeded axonal growth [15]. Supporting these observations, Park et al. [16] and Kim et al. [106] reported that LIPUS-treated groups exhibited larger axon diameters, thicker myelin sheaths, and more mature nerve structures, along with improved nerve conduction velocity. Additionally, Yonggang et al. [98] highlighted LIPUS-induced increases in angiogenesis, neurofilament density, and Tuj1 expression, indicating enhanced regeneration at both structural and molecular levels.

**Table 3:** Morphological and functional outcome of low-intensity pulsed ultrasound in vivo studies using complete injury model

Species	Model/Nerve	Parameters	Protocol	Follow-up (day)	Outcome	Reference
Wistar rat	Cut-sciatic nerve (neurotomized)	16 mW/cm^2^, 1.5 MHz	20 min/d, 12d	1d	- Led to rapid nerve regeneration post-axotomy - Morphological analysis showed increased metabolic activity in stimulated Schwann cells - The presence of newly formed vessels and organized myelin sheaths was observed	Crisci, 2002 [39]
SD rat	Cut-inferior alveolar nerve	30 mW/cm^2^, 1 MHz	20 min/day, 28d	28d	- Promoted recovery of sensory loss in facial skin above the mental foramen after IAN transection	Sato, 2016 [92]
SD rat	Cut+primary repair-median nerve	30 mW/cm^2^, 1.5 MHZ, (EXOGEN ultrasound)	every day, or 3d/week, or every week	56d	- The FDA-authorized ultrasound system failed to stimulate axonal regeneration because the ultrasound intensity was too low - However, it reduced muscle atrophy compared to sham-treated animals	Daeschler, 2018 [105]
SD rat	Nerve gap/10 mm autologous graft-sciatic nerve	250, 500, 750 mW/cm^2^, 1.0 MHz	5 min/day, every other day, 12d	84d	- Optimal LIPUS intensities for improving autograft nerve regeneration in rats - LIPUS at 250 mW/cm^2^ significantly enhances axonal regeneration rates - Excessive LIPUS can negatively impact recovery	Jiang, 2016 [94]
Lewis rat	Nerve gap/5 mm autologous graft-sciatic nerve	140 mW/cm^2^, 1.0 MHz	5 min/d, 1w or 4w	56d	In a rat autograft model, both one- and four-week ultrasound treatments support nerve reinnervation and suppress proinflammatory macrophage activity	Kawai, 2023 [100]
SD rat	Nerva gap/10 mm PLGA conduit seeded with SCs-sciatic nerve	200 mW/cm^2^, 1.0 MHz	5 min/session, daily, 12 sessions, 2w	42d	LIPUS activated the implanted-SCs in PLGA conduits to support nerve regeneration	Chang, 2004 [95]
SD rat	Nerve gap/15 mm PLGA conduit seeded with SCs-sciatic nerve	300 mW/cm^2^, 1.0 MHz	5 min/d, 12d	56d	- PLGA conduits show superior potential over silicone conduits for nerve tissue engineering - LIPUS supported better survival and proliferation of embedded SCs	Chang, 2005 [15]
SD rat	Nerve gap/12 mm PLGA/F127 conduit-sciatic nerve	400 mW/cm^2^, 1.0 MHz	2 min every week, 8w	56d	- Faster nerve regeneration rates with myelination and providing directional guidance to neurons - Demonstrated greater neural tissue growth, with enlarged axon diameter and enhanced myelin thickness - A synergistic interaction between this asymmetrically type of porous conduit and ultrasound therapy is suggested	Park, 2010 [16]
SD rat	Nerve gap/12 mm NGF-immobilized PCL-F127 conduit-sciatic nerve	400 mW/cm^2^, 1.0 MHz,	2 min/session, once/w, 24w	168d	- The NGF/US/NGC group exhibited superior nerve regeneration - Histological analysis revealed enlarged axon diameters and greater myelin layer thickness in the NGF/US/NGC cohort	Kim, 2013 [106]
SD rat	Nerve gap/12 mm iPSCs- NCSCs conduit-sciatic nerve	300 mW/cm^2^, 1.0 MHz,	5 min/d, 2w	90d	- LIPUS combined with iPSCs-NCSCs significantly boosted sciatic nerve function and conduction velocity - LIPUS treatment enhanced the density of nerve filaments and promoted neuron regeneration	Yonggang, 2015 [98]
SD rat	Nerve gap/10 mm PFTBA-GDF5, iPSCs–NCSCs conduit–sciatic nerve	300, 500 mW/cm^2^, 1 MHz	5, 10 min/d	90d	- LIPUS promotes proliferation and differentiation of iPSCs-NCSCs, enhancing nerve regeneration - PFTBA improves iPSCs-NCSCs viability under hypoxic conditions, aiding nerve repair - The combination of LIPUS, PFTBA, and GDF5 shows favorable effects on sciatic nerve injury repair	Xia, 2019 [99]

This table includes animal studies using more severe nerve injury models, including complete transection, surgical repair, autografting, or nerve guidance conduit implantation. It outlines the role of LIPUS in promoting regeneration, especially when combined with biomaterials, schwann cells, or growth factors

Beyond molecular signaling, extensive histological and immunohistochemical evidence demonstrates that LIPUS produces measurable structural improvements in regenerating nerves (Table 4). In vitro studies, LIPUS modulates key markers of SC and neuronal status: downregulation of P0 reflects a shift toward a proliferative SC phenotype conducive to axonal growth, whereas upregulation of Krox-20 supports transition back to a myelinating phenotype during remyelination [41]. Increased Dhh expression further prevents connective tissue encroachment into the nerve bridge [41]. Neuronal cytoskeletal maturation is reflected by enhanced (NF-M) and Tuj1 staining and corresponding qPCR upregulation, while elevated S100β and GFAP expression confirms SC maturation, cytoskeletal stability, and improved neurite support [88].

**Table 4:** Summary of histological endpoints used to evaluate LIPUS-induced peripheral nerve regeneration

Category	Markers/Techniques	Indications
Axonal Regeneration	NF-M, NF200	Maturation and density of regenerating axons; axonal integrity
	Belschowsky or toluidine stain	Overall neurofilament density in regenerating nerves, and axon counts
	Tuj1	Microtubule organization in neurons; neurite outgrowth
	MAP2	Neuronal differentiation and dendritic development
SC Activity	S100β	Mature Schwann cell marker; SC presence and supportive activity
	GFAP	Non-myelinating/pro-repair SC phenotype; early SC activation
	Krox20	Myelinating Schwann cell differentiation
	Dhh	SC signaling; preventing perineurial fibrosis and maintaining nerve structure
Remyelination	MBP	Myelin sheath formation and maturation
	P0	Major peripheral myelin protein; myelination status
	Myelin g-ratio	Degree of myelin maturity
	Osmium tetroxide staining	Myelin visualization for fiber density and morphometry
	Toluidine blue staining	Myelinated fiber classification, density, and distribution
Tissue Architecture	TEM	Myelin ultrastructure, axon area, fiber diameter, Myelin thickness, compact lamellae
	FESEM	Nanofiber alignment and extracellular matrix remodeling

This table compiles commonly used markers, staining methods, and structural readouts reported across in vitro and in vivo LIPUS studies. Endpoints include axonal regeneration, sc activity, myelin formation, and neuronal cytoskeletal changes

In vivo studies, these cellular effects translate into robust structural repair measurable across multiple modalities. Osmic acid and toluidine blue staining consistently reveal higher densities of myelinated fibers and more mature fiber-type distributions in LIPUS-treated nerves [39, 91, 96]. Transmission electron microscopy (TEM) shows thicker and more uniformly compact myelin sheaths, reduced g-ratios – the ratio of the inner axonal diameter to the total outer fiber diameter, larger axon diameters, increased axon area, and improved myelin-to-axon ratios, indicating accelerated axonal elongation and remyelination [16, 39, 44, 91, 94, 97, 106]. Field emission scanning electron microscopy (FESEM) imaging highlights improved nanofiber alignment and extracellular matrix organization in nerve conduits exposed to LIPUS [98]. Axonal regeneration is further validated by increased NF-M, NF-200, S100β [16, 99, 106], and Belschowsky-stained neurofilament density [98], while elevated microtubule-associated protein 2 (MAP2) and Tuj1 gene expression confirms enhanced neural differentiation at the injury site [99].

Together, these histological and ultrastructural findings demonstrate that the molecular and cellular responses induced by LIPUS—spanning SC activation, neuronal cytoskeletal remodeling, and modulation of the inflammatory and extracellular matrix environment—translate into quantifiable tissue-level improvements in axonal regeneration and remyelination across diverse nerve injury models. Moreover, during successful remyelination, regenerating axons typically exhibit a progressive decrease in g-ratio values, increased myelin sheath thickness, and the formation of compact, multilamellar myelin, which serve as standard histological indicators of functional nerve repair.

In terms of functional recovery, several preclinical studies have reported that LIPUS contributes to significant improvements in motor and sensory function. Functional assessments, including locomotor performance, limb posture, and electrophysiological responses compound muscle action potential (CMAP), were notably better in LIPUS-treated groups, as reported in numerous studies from 2015 to 2023 [94, 98, 100]. These results suggest that LIPUS supports both motor and sensory reinnervation. Notably, Kawai et al. [100] observed that even a short treatment duration (one week) produced significant CMAP improvements in autograft models. Complementing these motor benefits, Sato et al. [92] reported enhanced sensory recovery, with increased head-withdrawal thresholds and greater numbers of trigeminal ganglion neurons reinnervating facial skin after inferior alveolar nerve transection. Furthermore, studies by Yonggang et al. [98] and Xia et al. [99] demonstrated that nerve conduction velocity (NCV) approached near-normal levels in LIPUS-treated groups, especially when combined with stem cells or growth factors. While Daeschler et al. [105] and Kawai et al. [100] observed only partial improvements in motor outcomes or modest functional gains, both studies noted clear benefits of LIPUS, including reduced muscle atrophy and enhanced reinnervation.

Despite strong preclinical support for LIPUS, several studies have reported modest or inconsistent histological and functional outcomes, and these discrepancies can often be traced to methodological variability rather than biological limitations alone. Daeschler et al. found no significant improvements in grip strength, muscle atrophy, myelinated axon density, or CMAP amplitudes when using a median nerve transection and primary repair model—less commonly used than sciatic nerve models—and applied a human FDA-approved 30 mW/cm^2^ device [105], an intensity far below the 200–500 mW/cm^2^ range typically required to generate meaningful mechanobiological responses in rodent tissue [2]. Additionally, rats possess a short regeneration distance of the median nerve, and the authors sampled nerve segments only 6–8 mm distal to the lesion, a region where early regeneration may appear similar between groups, masking treatment effects of LIPUS. Their fixation and staining procedures, along with a custom semi-automated algorithm for axon quantification, introduce further variability that may influence axon counts – the total number of regenerated axons within a defined nerve segment and morphometric precision. In contrast, Kawai et al. used nerve grafts, which inherently vary in alignment and stability across animals, and applied 140 mW/cm^2^—an intensity appropriate for crush or simple transection [44, 91] but potentially insufficient for graft models with greater regenerative demands [100]. Importantly, histomorphometry was performed only at the graft midpoint, without assessing the injury site or distal stump, even though the authors noted that spontaneous regeneration in controls had already progressed by 8–18 weeks, likely diminishing observable differences. Despite improved CMAP amplitudes, histological improvements and functional recovery remained significantly unchanged, possibly due to axonal misdirection—an issue not easily captured by mid-graft sampling alone. Technical factors such as antibody selection, staining protocols, confocal imaging parameters, and batch variation may also contribute to inconsistent immunohistochemical outcomes. Collectively, these differences in model selection, sampling strategy, intensity choice, tissue processing, and analytical techniques highlight how methodological variability can obscure the true histological impact of LIPUS, underscoring the need for standardized, comparable, and scalable protocols with larger enough sample sizes in future studies. Addressing this heterogeneity will be critical to improving reproducibility and ensuring robust mechanistic understanding in the translation of LIPUS to clinical nerve repair. Collectively, these findings highlight the therapeutic potential of LIPUS in enhancing nerve signal transmission and promoting functional recovery, particularly when combined with advanced regenerative strategies.

## LIPUS induces neurotrophic factors and pro-inflammatory cytokines

Building upon the cellular and functional findings observed in both in vitro and in vivo studies, accumulating evidence highlights that the regenerative effects of LIPUS are mediated not only through direct stimulation of target cells such as neurons, Schwann cells, and neural stem cells but also through the modulation of key molecular signals that shape the repair microenvironment. Among these molecular mediators, neurotrophic factors and inflammatory cytokines play pivotal roles in coordinating axonal regrowth, remyelination, and tissue remodeling. The following section summarizes how LIPUS regulates these critical biochemical pathways to enhance peripheral nerve regeneration.

### Neurotrophic factors

A substantial body of preclinical research supports the conclusion that LIPUS enhances both the expression and activity of neurotrophic factors, which are critical mediators of neuronal survival, growth, and repair. Among these, NGF and BDNF are the most consistently upregulated factors across studies. For instance, Lu Zhao et al. [55] demonstrated that LIPUS significantly amplified NGF-stimulated neurite extension by enhancing the phosphorylation of ERK1/2 and CREB, and upregulating Trx-1, thereby promoting intracellular signaling pathways that support regeneration. Similarly, studies by Ren et al. [12], Siwak et al. [84], Wang et al. [91], and Kim et al. [106] showed that LIPUS stimulated SCs to secrete elevated levels of NGF, BDNF, GDNF, FGF, and NT3—all essential for axon guidance, remyelination, and target reinnervation. Further supporting evidence from Chen et al. [96] and Ni et al. [97] linked increased NGF and BDNF expression with improved neuronal and SC function. LIPUS also influenced neurotrophic factor expression in non-glial cells: Ventre et al. [89] observed NGF upregulation in sensory neurons, while Sato et al. [92] reported increased NT3 expression, which contributed to axonal elongation. Though Ito et al. [44] did not observe a significant NGF increase at one week post-injury, this was attributed to the timing of analysis rather than a lack of effect. Notably, Wang et al. [91] also found that LIPUS elevated VEGF-A alongside NGF and BDNF, suggesting a link between vascular support and neural recovery. Collectively, these findings emphasize that LIPUS promotes not only structural regeneration but also biochemical priming of the microenvironment through the robust stimulation of neurotrophic signaling, an essential mechanism for effective peripheral nerve repair.

### Other genes or proteins act as neurotrophic factors

In addition to enhancing well-established neurotrophic factors like NGF, BDNF, and NT3, LIPUS has been shown to modulate a broader array of non-traditional neuroregenerative molecules and signaling proteins that play essential roles in peripheral nerve healing. Tsuang et al. [41] reported that LIPUS downregulated pro-inflammatory genes such as IL-1, TNF-α, and P0, while upregulating Dhh, a critical factor in SC signaling and peripheral nerve morphogenesis. This molecular shift contributes to a more favorable microenvironment for SCs’ proliferation and axonal support. Similarly, Wen et al. [90] found that LIPUS stimulated the expression of netrin-1 and its receptor deleted in colorectal carcinoma, both essential for neuronal survival, axon guidance, and cell migration, suggesting enhanced axonal pathfinding through injury sites. Yonggang. Lv et al. [88] also observed increased expression of neuronal and glial markers, including NF-M, Tuj1, S100b, and GFAP, which are associated with neuronal integrity, glial support, and early-stage neural differentiation. Moreover, Xia et al. [99] highlighted that LIPUS can potentiate the regenerative effects of Growth Differentiation Factor 5 (GDF5), enhancing remyelination and nerve conduction, particularly when combined with supportive agents like perfluorotributylamine (PFTBA). Additionally, Sato et al. [92] reported that LIPUS upregulated VEGF in endothelial cells, reinforcing its role in promoting angiogenesis, which further supports nerve repair. Collectively, these findings emphasize that LIPUS acts beyond classic neurotrophic pathways, modulating a diverse spectrum of regenerative genes and proteins to orchestrate a more effective and multifaceted nerve healing process.

### Pro-inflammatory cytokines

Emerging evidence indicates that LIPUS modulates the pro-inflammatory cytokine expression, thereby influencing the viability and activity of SCs during peripheral nerve regeneration. Tsuang et al. [41] demonstrated that LIPUS temporarily downregulated TNF-α and IL-1 in serum-deprived SCs, followed by a delayed upregulation of IL-1. This suggests that LIPUS fine-tunes inflammatory responses over time to support SC survival and proliferation. This temporal modulation appears to be crucial, as Ren et al. [12] highlighted the roles of TNF-α, IL-1α, IL-1β, and MCP-1 in shaping the inflammatory environment and supporting SC viability. Additionally, Siwak et al. [84] demonstrated that LIPUS significantly reduced IL-1β and IL-6 expression in SCs-cytokines that, when overexpressed, are known to impair nerve repair. The suppression of these cytokines was even more pronounced when LIPUS was combined with pulsed electromagnetic fields, suggesting a synergistic anti-inflammatory effect. Further supporting these findings, Ito et al. [44] confirmed that LIPUS downregulated TNF-α and IL-6 expression during the acute post-injury phase, potentially facilitating more efficient Wallerian degeneration and establishing a regenerative environment for axonal regeneration. Together, these findings suggest that LIPUS not only promotes SC proliferation and function directly but also contributes to a pro-regenerative microenvironment by modulating inflammatory cytokine dynamics, an essential aspect of effective peripheral nerve healing.

## Dosage of LIPUS for peripheral nerve regeneration in preclinical in vivo studies

### Incomplete injury models

Across multiple preclinical studies using peripheral nerve crush injury models, LIPUS has been shown to significantly enhance nerve regeneration when delivered with carefully selected parameters. The applied LIPUS intensity typically ranged from 100 to 400 mW/cm^2^, with the effective outcomes observed between 140 and 250 mW/cm^2^. For example, Chen et al. [96] and Ni et al. [97] reported positive outcomes at 200–250 mW/cm^2^, while Ito et al. [44] identified 140 mW/cm^2^ as effective for promoting axonal sprouting and remyelination. Notably, Akhlagi et al. [101] tested 20 different parameter combinations and confirmed that non-heating, pulsed models ultrasound were more beneficial than continuous ones, with one group achieving up to 90% functional recovery within just 14 days.

The ultrasound frequency was most commonly set at 1.0 MHz [44, 91, 93, 96, 97, 101], although Mourad [102] successfully used 2.25 MHz and still observed accelerated functional recovery. Treatment duration per session typically ranged from 1 to 5 minutes, with session frequencies varying from daily, every other day, or three times per week. Chen et al. [96] and Ni et al. [97] applied LIPUS every other day, while Ito et al. [44] and Wang et al. [91] used daily protocols (five times per week), both reporting improved histological and functional outcomes. Total treatment durations spanned from 10 days [93] to 30 days [102], with longer regimens often resulting in more sustained and amplified regenerative benefits. Collectively, these studies show that moderate intensities and pulsed stimulation most effectively enhanced axonal growth, myelin thickness, and functional recovery, whereas continuous or excessive exposures were less beneficial.

### Complete injury models

Numerous preclinical studies of complete injury models using primary repair, autologous graft, and synthetic conduit have demonstrated that the effectiveness of LIPUS in enhancing peripheral nerve recovery depends heavily on the specific treatment parameters applied. The ultrasound frequency of LIPUS was uniformly set at 1.0 MHz across nearly all studies [15, 16, 92, 94, 95, 98–100, 106], confirming its standard use in neural tissue applications. Exceptions include Crisci et al. [39] and Daeschler et al. [106], who used a higher frequency of 1.5 MHz. Intensity levels varied widely, from as low as 16 mW/cm^2^ [39] to as high as 500 mW/cm^2^. However, the most consistent regenerative effects were observed within the range of 140–300 mW/cm^2^. Notably, Jiang et al. [94] reported that 250 mW/cm^2^ was more effective than higher intensities, suggesting that moderate intensities may be the better choice, maximizing efficacy while minimizing the risk of tissue overstimulation or damage. The duration of each treatment session ranged from 2 to 20 minutes, with 5 minutes per session being the most commonly used protocol in studies reporting positive histological and functional outcomes. The frequency of LIPUS application varied from daily treatments [15, 94, 95] to once-weekly sessions [106], with more frequent stimulation generally associated with faster and more consistent regeneration. In terms of total treatment duration, protocols ranged from short-term interventions lasting 5–12 days [15, 39] to extended regimens lasting up to 24 weeks [106]. Notably, beneficial effects, such as enhanced axonal elongation, reinnervation, and early functional recovery, were generally observed within the first 2 to 4 weeks of therapy. Together, these findings suggest that consistent, moderate-intensity pulsed stimulation applied over several weeks can effectively enhance both structural and functional outcomes in severe nerve injuries.

### Variabilities and challenges to future translation applications

Despite encouraging results, defining effective parameters for LIPUS in peripheral nerve regeneration remains a major challenge, as preclinical studies report considerable variability across species, injury models, and experimental designs. From the 20 in vivo studies summarized (Table 5), most used a frequency of 1 MHz (in 17 of 20 studies) and a 20% duty cycle, while intensity and treatment duration showed the widest variation and greatest influence on outcomes. Evidence consistently suggests that ultrasound intensity plays a more critical role than duration in determining biological responses. For example, Daeschler et al. [105] found that a clinically approved LIPUS device (30 mW/cm^2^) failed to promote axonal regeneration or functional recovery, likely because the intensity was below the cavitation threshold needed to trigger mechanobiological effects. In contrast, several studies employing moderate intensities (200–500 mW/cm^2^) reported enhanced Schwann cell proliferation, neurotrophin expression (NGF, ciliary neurotrophic factor), and myelination [2], even though most in vivo studies applied this range of intensities in 14 of 20 studies. Jiang et al. further observed that 250 mW/cm^2^ effectively promoted regeneration in autograft models, whereas higher intensities (≥500–750 mW/cm^2^) impaired outcomes, indicating a potential biphasic dose–response relationship [94]. Similarly, Kawai et al. found comparable reinnervation and macrophage modulation after one or four weeks of stimulation, suggesting that the duration of treatment may be less critical than the acoustic intensity [100]. Additional studies, including Mourad et al. and Ito et al., reported that moderate exposure levels (140–250 mW/cm^2^) yielded better histological regeneration and reduced pro-inflammatory cytokines (TNF-α, IL-6) and axonal inhibitors (SEMA3A, GSK3β), whereas higher intensities delayed recovery [44, 102]. Akhlagi et al. also demonstrated that lower intensities, shorter durations, and pulsed modes produced more favorable outcomes than continuous or high-intensity settings [101]. However, cross-study comparisons remain limited due to heterogeneity in species, nerve injury types (crush, transection, or autograft), sample sizes, evaluation endpoints, and follow-up durations, which complicates parameter standardization. Collectively, these findings emphasize that while intensity appears to be a key determinant of LIPUS efficacy, its optimal therapeutic range remains undefined. Future research should employ systematic, comparative experimental designs with standardized outcome measures to establish a reproducible framework for parameter selection and to clarify dose–response relationships across different nerve injury contexts.

**Table 5:** Summary of key LIPUS parameters, study models, and observed outcomes in preclinical in vivo studies on peripheral nerve regeneration

Species	N	Model-Nerve	Frequency MHz	Intensity mW/cm^2^	Duty cycle %	Protocol	Follow-up days	Outcome
NMRI mice	200	Crush-sciatic nerve	1.0, 3.0	200, 500, 1000	5, 20	2 min/day, daily, maximum 14d	14	- SFI was studied − 1 MHz was better, and duty cycle 20% was much more effective - Group US11 (1 MHz, 500 mW/cm^2^, 20%) showed significant difference in the final. [101]
Lewis rat	46	Crush-sciatic nerve	1.0	30, 140, 250	20	5 min/d, 5d/w, 1-3w	21	- Myelinated fiber density was significantly higher in 140 mW/cm^2^ (optimal intensity) - Suppressed pro-inflammatory and nerve growth inhibitor gene expression [44]
Lewis rat	90	Crush-sciatic nerve	1.0	140	20	5 min/d, daily in 14d and 5d/week, until death (30 days)	30	- Improved sciatic function, regeneration - upregulated growth factors and receptors [91]
Lewis rat	42	Crush-sciatic nerve	1, 2.25	250, 500, 1500	-	1 min/session, 3d/week, 30d	30	- Toe spread assay - The most successful protocol is 2,25 MHz, 250 mW/cm^2^ [102]
Wistar rat	20	Crush-sciatic nerve	1.0	400	20	2 min/d, 10d	21	- Improved SFI - Enhanced histo-morphologic properties [93]
Wistar rat	64	Crush-sciatic nerve	1.0	250	-	1 min/d, every other day, 8w	56	- Improved SFI, regeneration speed, and SNCV - Enhanced nerve fiber density [96]
SD rat	80	Crush-sciatic nerve	1.0	200	-	1 min/d, every other day	28	- Improved SFI, promoted axonal regeneration - Increased BDNF expression [97]
SD rat	40	Stretch-Brachial plexus (BPI)	1.0	100	20	20 min/d, 12d	28	- Enhanced functional recovery - Reduced neuroinflammation and spinal microglia overactivation [103]
SD rat	24	Crush-sciatic nerve	1.1	500	-	10 min/session, 3 sessions/w, 4w	56	- Improved SFI, muscle fiber diameter - Enhanced myelinated axons [104]
Wistar rat	35	Cut-sciatic nerve (neurotomized)	1.5	16	-	20 min/d, 12d	1	- Rapid nerve regeneration post-axotomy - Increased metabolic activity in stimulated SCs - Newly formed vessels and organized myelin sheaths [39]
SD rat	35	Cut-inferior alveolar nerve	1.0	30	-	20 min/day, 28d	28	- Promoted recovery of sensory loss [92]
SD rat	60	Cut+primary repair-median nerve	1.5	30	20	every day, or 3d/week, or every week	56	- failed to stimulate axonal regeneration, and function recovery - However, it reduced muscle atrophy [105]
SD rat	80	Nerve gap/10 mm autologous graft-sciatic nerve	1.0	250, 500, 750	20	5 min/day, every other day, 12d	84	- Improved SFI at 4w, best at low-dose - Enhanced axonal growth and regeneration at low-dose - Improved CMAPs at 3 months with low- and mid-dose - LIPUS at 250 mW/cm^2^ significantly enhances axonal regeneration rates - Excessive LIPUS (high-dose) negatively impact recovery [94]
Lewis rat	39	Nerve gap/5 mm autologous graft-sciatic nerve	1.0	140	20	5 min/d, 1w or 4w	56	- Improved CMAPs in for one or for 4 weeks of stimulation - No difference in motor function, histomorphometry, or muscle weight - Decreased number of pro-inflammatory macrophages - US therapy for one or 4 weeks can similarly promote reinnervation and reduce proinflammatory macrophages [100]
SD rat	52	Nerva gap/10 mm PLGA conduit seeded with SCs-sciatic nerve	1.0	200	20	5 min/session, daily, 12 sessions, 2w	42	- Statistically more myelinated axons with higher mean area of axons in US groups. [95]
SD rat	48	Nerve gap/15 mm PLGA conduit seeded with SCs-sciatic nerve	1.0	300	20	5 min/d, 12d	56	- Greater number and area of regenerated axons at the midconduit of implanted grafts in PLGA + US group [15]
SD rat	30	Nerve gap/12 mm PLGA/F127 conduit-sciatic nerve	1.0	400	20	2 min every week, 8w	56	- Faster nerve regeneration rates with myelination - Greater neural tissue growth, with enlarged axon diameter and enhanced myelin thickness [16]
SD rat	72	Nerve gap/12 mm NGF-immobilized PCL-F127 conduit-sciatic nerve	1.0	400	20	2 min/session, once/w, 24w	168	- The NGF/LIPUS/NGC group exhibited superior nerve regeneration - Enlarged axon diameters and greater myelin layer thickness in the NGF/US/NGC cohort [106]
SD rat	20	Nerve gap/12 mm iPSCs- NCSCs conduit-sciatic nerve	1.0	300	20	5 min/d, 2w	90	- Improved sciatic nerve function and conduction velocity in LIPUS + iPSCs-NCSCs - Enhanced density of nerve filaments and promoted neuron regeneration [98]
SD rat	12	Nerve gap/10 mm PFTBA-GDF5, iPSCs–NCSCs conduit–sciatic nerve	1.0	300, 500	20	5, 10 min/d	90	- Promoted proliferation and differentiation of iPSCs-NCSCs with LIPUS - PFTBA Improved iPSCs-NCSCs viability with PFTBA [99]

This table summarizes 20 in vivo studies examining the effects of LIPUS on peripheral nerve regeneration. It outlines key experimental parameters, frequency, intensity, duty cycle, session duration, total treatment period, and stimulation interval, along with corresponding animal models and injury types. The synthesis highlights how parameter selection influences regenerative outcomes and provides guidance for optimizing future preclinical and translational research

## Timing of applied LIPUS stimulation in peripheral nerve regeneration

Importantly, studies also highlighted the critical role of timing in LIPUS application. Crisci et al. [39] emphasized that applying LIPUS during the acute inflammatory phase post-injury resulted in more pronounced regenerative effects. Tsuang et al. [41] highlighted that early application of LIPUS, ideally within the first 8 hours post-injury, is crucial for maximizing its effects on SC proliferation. This early window aligns with a serum-deprived microenvironment at the injury site, where SCs are most responsive. Early application, at least within the first week, has a positive impact on macrophage activation and supports the overall nerve regeneration process [100]. This finding aligns with broader observations suggesting that early intervention enhances responsiveness to LIPUS, likely due to increased plasticity and receptiveness of the injured nerve microenvironment during the initial stages of healing.

## Other considerations beyond dosage parameters

Beyond dosage alone, therapeutic success with LIPUS is also influenced by tissue context and the presence of combinatorial treatment. Studies using NGCs seeded with SC [15, 95] or iPSC-derived stem cells [98, 99] demonstrated that LIPUS further enhanced outcomes such as myelination, angiogenesis, neural differentiation, and neurotrophic factor expression, particularly when paired with supportive agents like GDF5. These findings suggest that LIPUS does not act in isolation but interacts dynamically with the cellular and molecular components of the regenerative environment. As a result, optimal dosage selection should consider not only the injury model and timing of intervention but also the presence of scaffolds, stem cells, or biochemical cues. This context-dependent synergy underscores the importance of integrated therapeutic design to fully harness the regenerative potential of LIPUS.

## Role of LIPUS in modulating cellular signals for activation and proliferation – potential molecular mechanisms

**Fig. 5:**
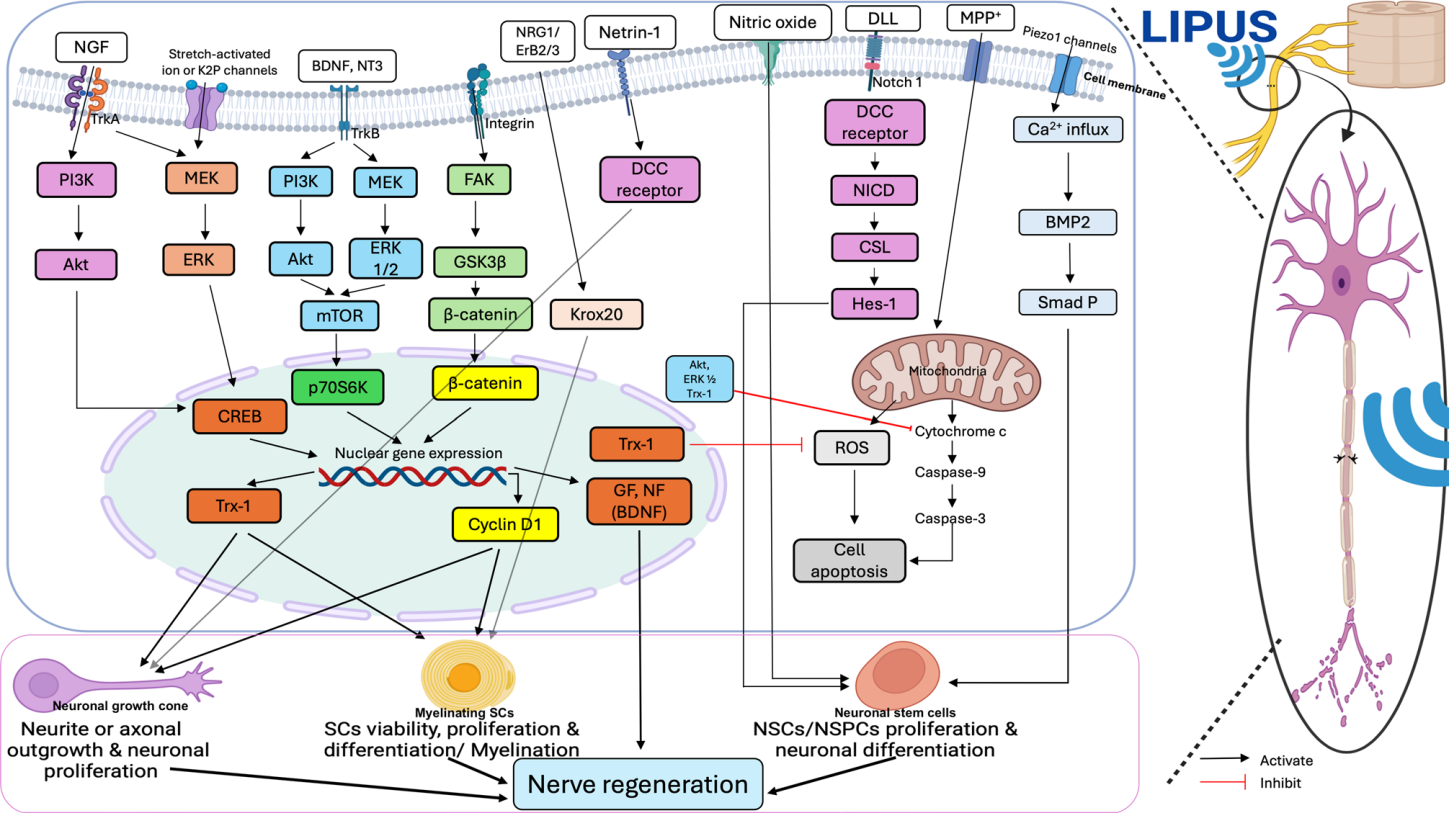
Integrated mechanobiological pathways regulated by LIPUS during peripheral nerve regeneration. The diagram illustrates how LIPUS-induced mechanical stimulation activates mechanosensitive receptors (integrins, Piezo1, and stretch-activated channels), triggering intracellular signaling cascades such as MAPK/ERK, PI3K/Akt, GSK3β/β-catenin, and BMP2/Smad. These pathways collectively promote neurite outgrowth, Schwann cell proliferation, remyelination, and neural stem cell differentiation, facilitating functional nerve recovery. LIPUS also upregulates key myelination-related molecules, including Krox20, myelin basic protein (MBP), and components of the NRG1/ErbB2/ErbB3 signaling pathway, indicating that NRG1/ErbB activation enhances sc proliferation, myelination, and neuroprotection. Additionally, the ERK1/2–CREB–Trx-1 axis supports neuronal survival, mitigates oxidative stress–induced apoptosis (MPP^+^ -induced cell death), and facilitates neurite elongation, underscoring the multifaceted molecular mechanisms through which LIPUS enhances peripheral nerve regeneration. Black arrows indicate activation; red lines indicate inhibition. PI3K: phosphatidylinositol-3-kinase; Akt: protein kinase B; MEK: mitogen-activated protein kinase/ERK kinase; erk: extracellular signal-regulated protein kinase; mTOR: mechanistic target of rapamycin; CREB: cAMP-regulated enhancer B; trx-1: thioredoxin-1; FAK: focal adhesion kinase; GSK3β: glycogen synthase kinase 3 beta; GF: growth factor; NF: neurotrophic factor; BDNF: brain-derived neurotrophic factor; NT-3: neurotrophin-3; NGF: nerve growth factor; DCC: deleted in colorectal carcinoma; DLL: delta-like; NICD: notch intracellular domain; CSL: CBF1, suppressor of hairless, lag-1; hes-1: hairy/enhancer of split 1; MPP+: 1-methyl-4-phenylpyridinium; ROS: reactive oxygen species; BMP2: bone morphogenetic protein 2; NSPCs: neural stem/progenitor cells; NSCs: neural stem cells; SCs: Schwann cells

The precise mechanisms by which LIPUS promotes peripheral nerve repair remain under active investigation. However, mounting evidence from both individual preclinical studies and review articles suggests a multifaceted network of mechanotransductive and molecular signaling pathways (Fig. 5). The therapeutic effects of LIPUS originate from its ability to convert physical acoustic energy, without thermal effect, into biochemical signals through a process known as mechanotransduction. As ultrasound waves propagate through biological tissue, they generate microscopic mechanical stresses, including pressure oscillations, acoustic streaming, and transient cavitation. These biomechanical forces deform cell membranes and extracellular matrices, activating mechanosensitive receptors, including integrins, Piezo1 channels, and stretch-activated ion channels. This physical-to-biological conversion initiates intracellular signaling cascades, including the PI3K/Akt, MAPK/ERK, and β-catenin pathways, which regulate cell proliferation, differentiation, and survival. Understanding this mechanobiological interface is therefore essential for linking ultrasound physics with its observed regenerative outcomes.

### Axonal or neurite outgrowth, neuronal viability, and proliferation

LIPUS promotes neurite extension and neuronal survival primarily through mechanotransduction-mediated activation of neurotrophic signaling pathways. Mechanical stimuli from LIPUS are sensed by stretch-activated ion channels and K2P channels, leading to Ca^2 +^ influx and activation of the TrkA receptor through enhanced NGF signaling. This triggers the phosphorylation of ERK1/2 and Akt, resulting in the activation of CREB and an increase in the expression of Trx-1—a multifunctional protein that acts as an antioxidant, neurotrophic cofactor, and suppressor of apoptosis. Together, the ERK1/2–CREB–Trx-1 axis supports neuronal viability, protects against oxidative stress (e.g., 1-methyl-4-phenylpyridinium (MPP^+^)-induced cell death), and promotes neurite elongation, as observed in PC12 and cortical neuron models [55, 107].

In addition, LIPUS enhances BDNF expression, which activates TrkB receptors, stimulating the PI3K/Akt and ERK/CREB pathways to further reinforce neuroprotection and neurite growth [108, 109]. Activation of the netrin-1/DCC signaling pathway contributes to neuronal survival by reducing apoptosis [90], while concurrent stimulation of mTOR signaling enhances protein synthesis and cytoskeletal dynamics required for axon elongation [86]. Collectively, these pathways form an integrated network that translates mechanical ultrasound cues into biochemical signals promoting axonal regeneration and neuronal resilience.

### Remyelination and Schwann cell viability, proliferation, and differentiation

In Schwann cells, LIPUS acts through integrin-dependent mechanotransduction to stimulate intracellular cascades governing survival, proliferation, and myelination. The primary signaling modules include the PI3K/Akt, GSK3β/β-catenin, and ERK/CREB–Trx-1 pathways. Upon LIPUS exposure, integrins and focal adhesions sense mechanical forces, leading to the phosphorylation of FAK and paxillin, which activates the PI3K/Akt signaling pathway [110–112]. This pathway supports SC survival, motility, and metabolic maintenance. Simultaneously, LIPUS induces FAK-mediated phosphorylation of GSK3β, stabilizing β-catenin and promoting its nuclear accumulation, which in turn upregulates cyclin D1 expression to drive SC proliferation and cell cycle progression [12, 113, 114].

Additionally, LIPUS enhances both ERK1/2 and Akt phosphorylation through TrkA activation induced by increased NGF expression, resulting in the phosphorylation of CREB and subsequent upregulation of Trx-1. Trx-1 functions as an antioxidant, neurotrophic cofactor, and anti-apoptotic protein, maintaining SC viability under stress conditions and supporting their regenerative phenotype [55]. Moreover, LIPUS upregulates Krox20, myelin basic protein, and the myelination-related NRG1/ErbB2/ErbB3 signaling pathway, suggesting that NRG1/ErbB activation contributes to SC proliferation, myelination, and the protective effects of LIPUS in peripheral nerve regeneration [115]. Through the integration of these signaling axes, LIPUS orchestrates a coordinated cellular response that enhances Schwann cell proliferation, differentiation, and remyelination, ultimately restoring nerve conduction and functional recovery.

### Neural stem cell proliferation and differentiation

LIPUS facilitates the proliferation and neuronal differentiation of neural stem cells (NSCs)/NSPCs via mechanosensitive calcium signaling and growth factor–mediated transcriptional regulation. Mechanical stimulation induces Ca^2 +^ influx through stretch-activated ion channels, leading to the activation of the ERK1/2 pathway and the upregulation of BDNF, which collectively promote neuronal differentiation [116]. LIPUS promotes neural stem cell (NSC) proliferation and neuronal differentiation through coordinated activation of the Notch and Piezo1–Ca^2 +^ – bone morphogenetic protein (BMP) 2/Smad pathways. By upregulating Notch1 and Hes1, LIPUS activates the canonical Notch cascade (Notch receptors, Jagged/Delta ligands, CSL- CBF1, suppressor of hairless, lag-1), balancing NSC proliferation while favoring neuronal over glial differentiation. Concurrently, LIPUS-induced deformation of the F-actin cytoskeleton activates Piezo1 channels, triggering Ca^2 +^ influx, BMP2 expression, and Smad phosphorylation, which drive transcription of neuronal differentiation genes. These synergistic mechanosensitive processes convert acoustic stimulation into biochemical signaling that enhances neurogenesis and functional maturation of NSCs [85, 117]. Moreover, LIPUS exposure increases cell membrane permeability and enhances Ca^2 +^ influx, which stimulates nitric oxide (NO) release. The elevated NO levels then promote the transition of NSPCs from proliferation to neuronal differentiation [43, 118]. By stimulating these coordinated molecular events, LIPUS effectively enhances the regenerative potential of stem cell-derived neural populations, supporting their integration into injured nerve environments and contributing to long-term functional repair.

In summary, LIPUS exerts its regenerative effects through a multifactorial mechanobiological framework. By converting acoustic energy into targeted molecular signaling, it activates key pathways—ERK1/2–CREB–Trx-1, PI3K/Akt, GSK3β/β-catenin, Notch, and BMP2/Smad—that regulate neuronal survival, SC proliferation, and NSC differentiation. The convergence of these cascades promotes axonal elongation, remyelination, and functional recovery, underscoring the broad therapeutic potential of LIPUS in peripheral nerve regeneration. The molecular mechanisms activated by LIPUS, such as enhanced Schwann cell proliferation, neurotrophin secretion, and modulation of inflammatory signaling, translate directly into the structural and functional improvements observed in preclinical models. For example, activation of the ERK1/2–CREB axis promotes axonal elongation, while PI3K/Akt signaling supports neuronal survival and remyelination. These coordinated cellular events collectively contribute to accelerated axonal regrowth, increased myelinated fiber density, and improved electrophysiological recovery demonstrated across animal studies. Bridging these mechanistic insights with experimental outcomes highlights how LIPUS exerts multifaceted benefits on peripheral nerve regeneration, guiding its potential translation into clinical applications.

### Further well-designed molecular and cellular studies for undetermined specific factors

Despite significant advances in elucidating LIPUS-induced signaling cascades, several critical cellular and molecular mechanisms remain poorly understood. The primary mechanosensors responsible for converting acoustic forces into biochemical signals in neurons, SCs, and NSCs are not yet clearly identified. While channels such as Piezo1/2, ion channels are potential candidates, their relative contributions and downstream effects require systematic validation. The role of nuclear mechanotransducers, particularly the YAP/TAZ–Hippo pathway, also remains unexplored, although it likely regulates SC proliferation and stem cell fate decisions. Furthermore, the cavitation threshold and membrane sonoporation dynamics that initiate intracellular signaling have not been quantitatively characterized in neural tissues. LIPUS-induced extracellular vesicle (exosome) release and microRNA-mediated communication represent additional, largely unexplored layers of intercellular regulation. Moreover, the potential influence of mitochondrial metabolism, oxidative stress modulation, and calcium-dependent transcriptional programs on LIPUS outcomes remains to be clarified. Finally, the integration of these pathways through epigenetic and transcriptional reprogramming mechanisms, including chromatin remodeling and noncoding RNA networks, is still unknown. Addressing these mechanistic gaps will be essential for defining the precise molecular hierarchy of LIPUS action and optimizing its therapeutic application for nerve repair.

## Current limitations and clinical perspective of LIPUS in peripheral nerve regeneration

### Current limitations in the previous preclinical studies

Although LIPUS shows considerable promise in promoting peripheral nerve regeneration, several limitations remain. One major challenge is the lack of standardized ultrasound parameters, which hampers cross-study comparison and reproducibility [12, 15, 55]. While in vitro models offer valuable mechanistic insights, they fail to fully replicate the complex in vivo environment, limiting translational relevance [41, 84]. Furthermore, many studies focus on short-term outcomes, with long-term functional recovery and chronic inflammatory modulation remaining underexplored [85, 90]. Mechanistic understanding is also incomplete. Although pathways such as ERK1/2–CREB–Trx-1 [55], GSK-3β/β-catenin [12], and netrin-1/DCC [90] have been implicated, the precise molecular link between LIPUS-induced mechanical stimulation and downstream intracellular signaling remains unclear. Small sample size and the use of short nerve gap models further reduce statistical power and clinical applicability [94]. In addition, most research has concentrated on SCs and neurons, with relatively little attention paid to other critical cell types such as fibroblasts, macrophages, and endothelial cells [92, 100]. Hypoxic conditions in graft environments also pose challenges for stem cell survival and differentiation [99]. Notedly, temperature-mediated effects of ultrasound are not always explicitly ruled out in experimental designs, raising concerns about potential unintended bioeffects [44]. To address these limitations, future studies should focus on optimizing LIPUS parameters across various biological contexts and developing more clinically relevant models of chronic or long-gap nerve injury. Functional evaluations should be extended beyond motor recovery to include sensory and behavioral outcomes [16, 102]. Deeper molecular profiling using high-throughput methods is warranted to elucidate underlying mechanisms, and enhanced biomaterials and oxygenation strategies are needed to improve stem cell-based therapies [98, 99]. Ultimately, large-scale, rigorously controlled in vivo studies will be essential to validate LIPUS as a clinically viable modality for peripheral nerve repair.

### Clinical translation barriers and future perspective

Preclinical studies increasingly support the therapeutic potential of LIPUS for PNI, demonstrating its ability to enhance SC viability, promote neural stem cell differentiation, and facilitate neurite outgrowth, effects mediated in part through nitric oxide signaling and neurotrophic factor pathways [12, 15, 41, 43, 55, 85, 88]. When combined with agents such as NGF or methylcobalamin, LIPUS has shown synergistic effects, accelerating axonal regeneration and improving motor function in challenging models such as BPI [55, 103]. These findings suggest that LIPUS could serve as an effective adjunct to pharmacological or scaffold-based therapies.

Innovative approaches, including LIPUS-mediated modulation of SC-derived exosomes and microRNAs, further broaden its therapeutic scope, particularly for complex conditions such as neurodegeneration and neurotrauma [83, 86]. In vivo studies consistently show that LIPUS promotes reinnervation, reduces inflammation, and enhances functional recovery across various injury models—including nerve crush, transection, or grafting [44, 91, 93, 96, 100, 102]. Moreover, integration of LIPUS with nerve conduits or autografts significantly improves regeneration outcomes [16, 95, 98, 99, 106], supporting its role in multimodal repair strategies.

The recent concept of remote LIPUS, in which ultrasound is applied to a site distant from the actual nerve injury yet still results in improved regeneration, presents an exciting opportunity for non-contact, broad-access therapy. This may be especially valuable in clinical scenarios where direct access to injured nerves is not feasible [104].

Although extensive preclinical evidence supports the efficacy of LIPUS in various PNI models, including crush, transection, primary repair, autograft, and conduit reconstruction, its application remains largely confined to these controlled experimental paradigms. To achieve clinical translation, further studies must extend to more complex and clinically relevant scenarios, such as nerve transfer procedures and proximal injuries, where LIPUS could potentially enhance axonal regeneration, reinnervation, and functional recovery. However, several key barriers currently limit clinical adoption.

First, regulatory classification poses a significant hurdle, as existing FDA-approved LIPUS devices are only indicated for bone healing, requiring new safety and efficacy validation for neural applications. Second, parameter standardization remains unresolved; variability in intensity, frequency, duty cycle, and exposure duration across preclinical studies complicates the identification of safe and effective clinical settings. Moreover, anatomical differences between animal models and humans, including nerve depth, tissue composition, and fascicular complexity, influence energy delivery and may necessitate device recalibration. Patient-specific factors such as age, metabolic status, comorbidities, and injury severity further contribute to inter-individual variability in therapeutic response.

Practical barriers also persist, including maintaining consistent transducer placement, ensuring treatment compliance, and integrating LIPUS into postoperative rehabilitation workflows. Additionally, maintaining effective blinding and placebo control in clinical trials remains challenging due to the perceptible acoustic and tactile cues associated with ultrasound. On the mechanistic side, human evidence confirming molecular activation pathways, such as ERK1/2, β-catenin, and mechanosensitive ion channel signaling, remains limited. The long-term safety of repetitive neural exposure also requires further validation. Excessive or prolonged stimulation may even risk tissue overstimulation or impaired recovery. Finally, economic and regulatory considerations, including the absence of reimbursement frameworks and cost-effectiveness analyses, present further obstacles to routine clinical use.

Future research should prioritize standardized dosimetry, optimized device design, and multicenter clinical trials with consistent endpoints to establish reproducible data on safety and efficacy. Integrating real-time imaging and feedback systems could also enhance treatment precision. Addressing these translational challenges will be essential for positioning LIPUS as a safe, noninvasive, and practical adjunct to surgical nerve repair and emerging regenerative strategies.

## Conclusion

LIPUS has emerged as a promising, noninvasive neuromodulatory therapy capable of enhancing peripheral nerve regeneration through both cellular and molecular mechanisms. Preclinical evidence consistently demonstrates that LIPUS promotes Schwann cell proliferation, supports neuronal survival, and stimulates neural stem cell differentiation by activating key mechanobiological signaling cascades, including ERK1/2–CREB–Trx-1, PI3K/Akt, GSK3β/β-catenin, Notch, and BMP2/Smad. These pathways collectively drive axonal elongation, remyelination, and neurotrophic factor upregulation, contributing to structural and functional recovery following nerve injury.

Despite substantial progress, translation to clinical practice remains constrained by heterogeneity in experimental parameters, species-specific responses, and limited long-term safety and mechanistic validation in humans. Key challenges include defining effective and reproducible dosage parameters, overcoming anatomical and physiological differences between animal and human models, and addressing regulatory and logistical barriers to clinical implementation.

Future efforts should focus on standardizing LIPUS dosimetry, employing clinically relevant large-animal and chronic injury models, and integrating advanced imaging and feedback systems for precise energy delivery. Moreover, elucidating unresolved mechanosensitive signaling events, such as the role of Piezo channels, YAP/TAZ mechanotransduction, and exosome-mediated communication, will be essential for refining therapeutic strategies. Through collaborative, multicenter studies and optimized device design, LIPUS holds strong potential to evolve from a preclinical modality into a clinically viable adjunct for enhancing nerve repair, functional restoration, and patient recovery.

## Data Availability

All data generated or analyzed in this study are available from the corresponding author upon reasonable request.

## Abbreviation

Akt: Protein kinase B
BDNF: Brain-derived neurotrophic factor
BMP: Bone morphogenetic protein
BPI: Brachial plexus injury
cAMP: cyclic adenosine monophosphate
CCL: C-C motif chemokine ligand.
CMAP: Compound muscle action potential
CNS: Cranial nervous system
CREB: cAMP-regulated enhancer B
CSL: CBF1, suppressor of hairless, lag-1
CTS: Carpal tunnel syndrome.
DCC: Deleted in colorectal carcinoma
Dhh: Desert Hedgehog
DLL: Delta-like
DRG: Dorsal root ganglia
ECM: Extracellular matrix
ERK: Extracellular signal-regulated protein kinase
FAK: Focal adhesion kinase
FESEM: Field emission scanning electron microscopy
FGF: Fibroblast growth factor
FoxO: forkhead box O
GAP-43: Growth-associated protein-43
GDF5: Growth differentiation factor 5
GDNF: Glial cell line-derived neurotrophic factor
GFAP: Glial fibrillary acidic protein
GSK3β: Glycogen synthase kinase 3 beta
Hes-1: Hairy/enhancer of split 1
IL-: Interleukin-
iPSCs: Induced pluripotent stem cells
iPSC-NCSCs: neural crest stem cells derived from induced pluripotent stem cells
JAK/STAT: Janus kinases/Signal transducers and activators of transcription
LIF: Leukemia inhibitory factor
LIPUS: Low-intensity pulsed ultrasound
MAP2: Microtubule-associated protein 2
MAPK: Mitogen-activated protein kinase
MCP-1: Monocyte chemoattractant protein-1
MEK: Mitogen-activated protein kinase/ERK kinase
MPP+: 1-methyl-4-phenylpyridinium
Mtor: Mechanistic target of rapamycin
NCV: Nerve conduction velocity
NF-M: Neurofilament medium chain
NF200: Neurofilament 200
NGCs: Nerve guidance conduits
NGF: Nerve growth factor
NICD: Notch intracellular domain
NMJ: Neuromuscular junction
NSC: Neural stem cell, NSCs: Neural stem cells
NSPCs: Neural stem/progenitor cells
NT-3: Neurotrophin-3
P0: Protein zero
PCL-F127: Polycaprolactone/Pluronic F127
PFTBA: Perfluorotributylamine
PI3K: Phosphoinositide 3-kinase
PICO: Population, intervention, comparison, outcome
PKA: Protein kinase A
PKC: Protein kinase C
PLGA: Poly(lactic-co-glycolic acid)
PNI: Peripheral nerve injury
PRISMA: Preferred reporting items for systematic reviews and meta-analyses
ROS: Reactive oxygen species
SC: Schwann cell
SCs: Schwann cells
SEMA3A: Semaphorin 3A
SFI: Sciatic functional index
TEM: Transmission electron microscopy
TNF-α: Tumor necrosis factor-alpha
Trk-A, Trk-B: Tropomyosin receptor kinase A, B
Trx-1: Thioredoxin-1
Tuj1: Class III β-tubulin
VEGF: Vascular endothelial growth factor
YAP/TAZ-TEAD1: Yes associated protein/Transcriptional co-activator with PDZ-binding motif- TEA domain transcription factor 1
